# BMI trajectories from birth to young adulthood associate with distinct cardiometabolic profiles

**DOI:** 10.1186/s12916-024-03741-0

**Published:** 2024-11-05

**Authors:** Gang Wang, Dang Wei, Simon Kebede Merid, Sandra Ekström, Susanna Klevebro, Natalia Hernandez-Pacheco, Sophia Björkander, Petter Ljungman, Inger Kull, Jochen M. Schwenk, Anna Bergström, Erik Melén

**Affiliations:** 1 Division of Internal Medicine, Institute of Integrated Traditional Chinese and Western Medicine, West China Hospital, Sichuan University, Chengdu, Sichuan China; 2 Department of Clinical Science and Education, Södersjukhuset, Karolinska Institutet, 118 83 Stockholm, Sweden; 3 Institute of Environmental Medicine, Karolinska Institutet, Stockholm, Sweden; 4 West China Biomedical Big Data Center, West China Hospital, Sichuan University, Chengdu, China; 5 Centre for Occupational and Environmental Medicine, Region Stockholm, Stockholm, Sweden; 6 Sachs’ Children and Youth Hospital, Södersjukhuset, Stockholm, Sweden; 7 CIBER de Enfermedades Respiratorias (CIBERES), Instituto de Salud Carlos III, Madrid, Spain; 8 Department of Cardiology, Danderyd Hospital, Stockholm, Sweden; 9 Science for Life Laboratory, Department of Protein Science, KTH Royal Institute of Technology, Stockholm, Sweden

**Keywords:** Bioimpedance, Childhood, Inflammation, Lipid, HbA1c

## Abstract

**Background:**

Numerous studies have investigated links between body mass index (BMI) trajectories and cardiovascular risk, yet discrepancies in BMI measurement duration and timing of the cardiovascular-related outcome evaluation have led to inconsistent findings.

**Methods:**

We included participants from the Swedish birth cohort (BAMSE) and applied latent class mixture modeling to identify BMI trajectories using data of multiple BMI measures (≥ 4 times) from birth until 24-year follow-up (*n* = 3204). Subsequently, we analyzed the associations of BMI trajectories with lipids (*n* = 1974), blood pressure (*n* = 2022), HbA1c (*n* = 941), and blood leukocytes (*n* = 1973) using linear regression. We also investigated the circulating levels of 92 inflammation-related proteins (*n* = 1866) across BMI trajectories.

**Results:**

Six distinct BMI groups were identified, denoted as increasing—persistent high (*n* = 74; 2.3%), high—accelerated increasing (*n* = 209; 6.5%), increasing—accelerated resolving (*n* = 142; 4.4%), normal—above normal (*n* = 721; 22.5%), stable normal (*n* = 1608; 50.2%), and decreasing—persistent low (*n* = 450; 14.1%) BMI groups. The increasing—persistent high and high—accelerated increasing BMI groups had higher levels of total cholesterol [mean difference (95% confidence intervals): 0.30 (0.04–0.56) and 0.16 (0.02–0.31) mmol/L], triglyceride, low-density lipoprotein, hemoglobin A1C [3.61 (2.17–5.54) and 1.18 (0.40–1.98) mmol/mol], and low-density lipoprotein/high-density lipoprotein ratios, but a lower level of high-density lipoprotein than the stable normal BMI group. These two groups also had higher leukocyte cell counts and higher circulating levels of 28 inflammation-related proteins. No increased cardiometabolic markers were observed in the increasing—accelerated resolving BMI group.

**Conclusions:**

Participants with persistently high or accelerated increasing BMI trajectories from birth to young adulthood have elevated levels of cardiometabolic risk markers at young adulthood than those with stable normal BMI. However, a raised BMI in childhood may not be inherently harmful to cardiometabolic health, provided it does not persist into adulthood.

**Supplementary Information:**

The online version contains supplementary material available at 10.1186/s12916-024-03741-0.

## Background

Obesity has become more prevalent in the past decades [1] and is a strong risk factor for cardiovascular diseases (CVD) [2]. Obesity is considered a state of low-grade chronic inflammation [3] and is associated with insulin resistance [2]. Indeed, obesity may accelerate the atherosclerotic process, that is generally initiated in early life, through these mechanisms [2]. Moreover, excess adiposity can activate the renin–angiotensin–aldosterone and sympathetic nervous systems, contributing to high blood pressure and dyslipidemia [2, 4] Obesity can also result in myocardial fat accumulation and fibrosis, in turn leading to left ventricular remodeling and subsequent left ventricular dysfunction, atrial fibrillation, and heart failure [2].

Body mass index (BMI) is a commonly used measurement to assess adiposity in routine clinical practice and research. Longitudinal information on BMI can capture dynamic shifts over time, such as the age of obesity onset and its progression. Numerous studies have found that certain BMI trajectories are associated with CVD and cardiovascular risk factors [5–8]. Particularly, some cohort studies have focused on BMI trajectories starting from childhood and the association with cardiometabolic risk in later life [9–16].

However, birth weight and BMI development in early life are associated with subsequent CVD [15]. Thus, BMI trajectories from birth are presumably sensitive to identifying individuals with a high risk of CVD in later life. Although several previous studies have attempted to assess BMI trajectories from birth [9–13], outcomes were typically assessed in early adolescence when cardiometabolic profiles often have not yet exhibited significant differences. In addition, most prior studies had limited sample sizes, which were not powered enough to identify specific BMI trajectories and their associations with subsequent outcomes. Therefore, there is a clear need for studying BMI trajectories from birth throughout childhood and their association with cardiovascular risk in a large longitudinal cohort with available information throughout childhood, starting from birth.

To this end, we conducted a longitudinal cohort with multiple BMI measurements from birth and a range of cardiometabolic profiles assessed at early adulthood to investigate BMI trajectories from birth and the association with cardiometabolic risk. In addition, we performed a comprehensive literature review on published studies to date that applied latent class group modeling or equivalents to identify BMI trajectories and assessed associations with cardiovascular risk in early or middle adulthood.

## Methods

### Study population

The Swedish population-based birth BAMSE (Swedish abbreviation for Child [Barn], Allergy, Milieu, Stockholm, Epidemiological) cohort recruited 4089 infants from inner-city, urban, and suburban districts of Stockholm, Sweden, between February 1994 and November 1996 and followed them from birth until 26 years (with questionnaires and/or clinical visit follow-up at 1, 2, 4, 8, 12, 16, 24, and 26 years) [17]. The study was approved by the Regional Ethical Review Board in Stockholm (Ref 2016/1380–31/2). The parents and participants signed their informed consent, under the Helsinki Declaration.

### Assessment of body mass index

We retrieved information on birth weight and length from the Swedish Medical Birth Register and collected weight and height from school and healthcare records at ages around 6 (± 2 weeks), 12, and 18 months (± 4 weeks), as well as 2, 3, 4, 5 (± 6 months), 7, 10, and 12 years (− 6 to + 11 months) [18]. Moreover, clinical assessments, including BMI, were carried out at around age 4, 8, 16, and 24 years following standardized protocols at each visit by trained nurses during the follow-up. Furthermore, self-reported weight and height data were also collected at recruitment and ages 12, 16, and 24 years. The hierarchy for utilizing height and weight information prioritized data from clinical investigations and the Swedish Medical Birth Register, followed by school and healthcare records, with self-reported data being considered last (Additional file 1: Table S1).

BMI was calculated as weight in kilograms divided by the square of height in meters (kg/m^2^). Subsequently, it was standardized into z-scores employing the World Health Organization child growth standards for ages 0–5 years [19] and growth reference data for those aged 5–19 years [20]. At the age of 24 years, BMI was transformed into sex-specific BMI z-scores based on the observed values within our cohort. We only included participants with at least four BMI measurements in this analysis, leading to a total of 3204 participants (Additional file 2: Fig. S1) with a mean of 10.5 BMI measurements (interquartile range: 8–13). Besides, we conducted a sensitivity analysis to compare BMI differences between participants with and without BMI data at 24 years of age. Additionally, bioimpedance measurements were conducted using the Tanita MC 780 body composition monitor (Tanita Corp., Tokyo, Japan) at the clinical follow-up of age 24 and 26 years, following the manufacturer’s guidelines (*n* = 1958 and *n* = 931, respectively). We derived fat mass index (FMI) and fat-free mass index (FFMI), calculated as masses in kilograms divided by the square of height in meters (kg/m^2^).

### Assessment of cardiometabolic profiles

Resting systolic and diastolic blood pressure was measured at 24 years of age (*n* = 2022) by trained nurses using standardized protocols and an automatic blood pressure meter (Omron HBP–1300, Omron Electronics, Kyoto, Japan). Blood pressure was assessed three times for each participant, with a 1-min pause between measurements. The analysis incorporated the mean blood pressure derived from all three measurements for each participant.

Blood samples collected at the clinical follow-up at age 24 years were used to quantify blood lipid levels by Karolinska University Laboratory (Stockholm, Sweden), including triglyceride (TG), total cholesterol, low-density lipoprotein (LDL), and high-density lipoprotein (HDL) (*n* = 1974). The levels of 92 inflammation-related proteins (Additional file 1: Table S2) were analyzed in ethylenediaminetetraacetic acid (EDTA) plasma samples from 1866 participants included in the current study (Inflammation Panel version 95,302, Olink Proteomics, Uppsala, Sweden). Details of the protein measurement have been described previously [17]. Additionally, we also assessed hemoglobin A1C (HbA1c) levels in blood among 941 participants at the clinical follow-up of age 26 years at Karolinska University Laboratory (Stockholm, Sweden). In addition, clinical-related thresholds were utilized to delineate heightened cardiometabolic risks (details in Additional file 3) [21–23].

### Statistical analyses

#### Latent BMI trajectories identification

Latent class mixture modeling (LCMM) was employed to investigate the longitudinal progression of BMI z-scores from birth to age 24 years. Several criteria, such as the mean absolute error loss, the Bayesian Information Criterion (BIC), log-likelihood, and the values of mean posterior class membership probabilities, and clinical plausibility were used to select the BMI trajectories. Additional file 3 offers full details regarding the methodologies employed for model construction and the determination of the optimal number of trajectories [24, 25].

#### Associations of BMI z-scores trajectories with cardiometabolic profiles

Covariates were compared between different BMI z-scores trajectories using *t*-test/ANOVA, Kruskal–Wallis rank sum test, chi-squared tests, and Fisher’s exact test as appropriate. We examined the associations of BMI trajectories with blood pressure, blood lipid, leukocytes, and HbA1c using multivariable linear regression models (details in Additional file 3) [26]. Besides, logistic regression was used to examine the association of BMI trajectories with any cardiometabolic risk. In addition, we performed a stratified analysis by sex to assess the differences in the associations between males and females. Sensitivity analyses were conducted for cardiometabolic profiles in early adulthood, incorporating BMI z-scores at birth, BMI z-scores at the 24 years, or the fat mass index into the regression models, respectively.

Protein concentrations were normalized based on inverse normal transformation [27]. We first compared plasma inflammation-related protein levels across BMI trajectories using ANOVA. We then employed multivariable linear regression to analyze the associations of BMI trajectories with proteins with significant associations with BMI trajectories (*p* < 0.05) (Additional file 1: Table S3). Multiple comparisons were corrected by applying Benjamini-Hochberg’s method [28]. We examined the protein associated with cardiometabolic profiles for their expression patterns in tissues and cells [29] by using the Human Protein Atlas (https://www.proteinatlas.org).

All the analyses were performed using R 4.2.2.

## Results

### BMI trajectories from birth to young adulthood

**Fig. 1:**
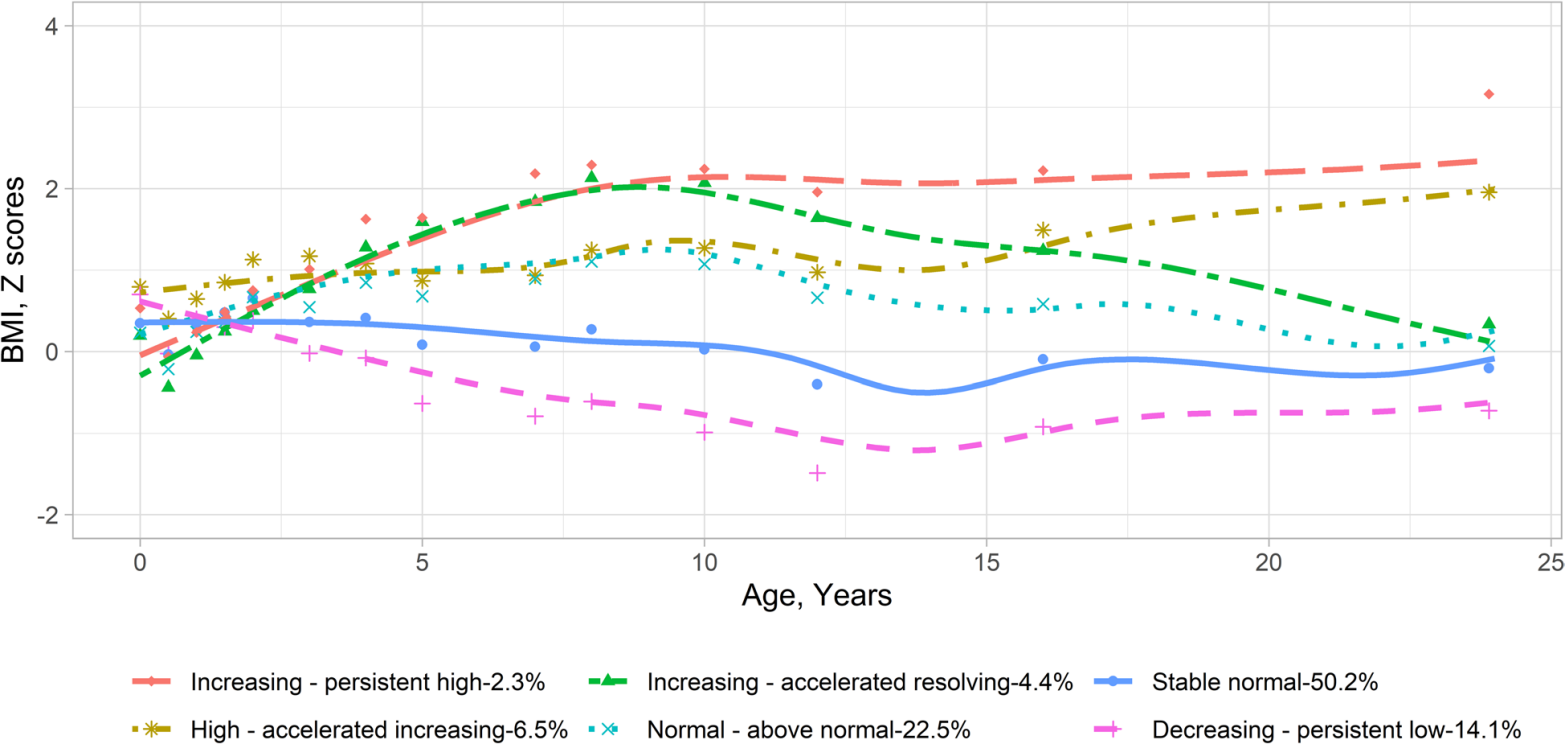
Body mass index trajectories from birth to young adulthood in the BAMSE cohort. Body mass index trajectories were identified through latent class mixture models using z-score of body mass index. The dots show the mean values for body mass index z-scores around each follow-up point. The lines show the loess-smoothed body mass index trajectories for the six identified trajectory groups. The statistics (mean and standard deviation of BMI in each group) of this figure are presented in Table S7. BMI, body mass index

Participants included in the BMI trajectory analysis showed, compared to those excluded, higher parental education levels, less maternal smoking during pregnancy, older maternal delivery age, lower parity before the index person was born, and more exclusive breast feeding (Additional file 1: Table S4). To explore potential BMI trajectories in BAMSE, we initially compared models with various terms using mean absolute error loss from cross-validation. We found that LCMM models with quadratic terms showed greater predictive accuracy compared to linear ones, functioning similarly to those with cubic terms (Additional file 1: Table S5). Subsequently, we identified distinct BMI trajectories based on criteria such as BIC, log-likelihood, mean posterior class membership probabilities > 0.7, and clinical plausibility (Additional file 1: Table S6). Consequently, our analysis revealed six trajectories, which included (1) the increasing—persistent high BMI group (*n* = 74 [2.3%])—BMI z-score was normal in early life, increased during childhood, and kept high in adolescence; (2) the high—accelerated increasing BMI group (*n* = 209 [6.5%])—BMI z-score was high at early life, kept high during childhood, and increased during adolescence; (3) the increasing—accelerated resolving BMI group (*n* = 142 [4.4%])—BMI z-score was low at early life, increased sharply during childhood, but decreased in adolescence; (4) the normal—above normal BMI group (*n* = 721 [22.5%])—BMI was normal at the early life and increased above the average level but within the normal range throughout childhood and adolescence; (5) the stable normal BMI group (*n* = 1608 [50.2%])—BMI z-score was consistently normal from birth until young adulthood; and (6) the decreasing—persistent low BMI group (*n* = 450 [14.1%])—BMI z-score was normal at the early life and decreasing to lower than average and keep low in adolescence (Fig. 1 for BMI z-scores, Additional file 2: Fig. S2 for raw BMI values, and Additional file 1: Table S7 for statistics). In the sensitivity analysis, participants with BMI data at 24 years showed lower BMI z-scores at specific time points compared to those without BMI data at 24 years. These differences were observed at 12 and 16 years in the increasing—persistent high BMI group; at 6 months, 1 year, 12 years, and 16 years in the high—accelerated increasing BMI group; and at 6 months, 1 year, and 16 years in the normal—above normal BMI group (Additional file 2: Fig. S3). Similar patterns of such differences were observed among BMI values (Additional file 2: Fig. S4).

### Associations of BMI trajectories with cardiometabolic profiles in young adulthood

Higher proportion of males was observed in the increasing—accelerated resolving BMI group and the normal—above normal BMI group than in the stable normal BMI group. Participants in the increasing—persistent high, increasing—accelerated resolving, and normal—above normal BMI groups were more likely to be born prematurely, by cesarean section, and to mothers with hypertension during pregnancy than those in the stable normal BMI group. Participants in the decreasing—persistent low BMI group were more likely to be born to mothers with low BMI at early pregnancy than those in the stable normal BMI group, whereas the other groups had mothers with higher BMI at early pregnancy. Additionally, participants in the high—accelerated increasing, increasing—accelerated resolving, and normal—above normal BMI groups were more likely to be born to mothers who smoked during early pregnancy (Table 1). Similar patterns of such differences between BMI subgroups were observed among males and females (Additional file 1: Tables S8 and S9). Participants in the increasing—persistent high, high—accelerated increasing, and increasing—accelerated resolving BMI group had higher FMI and FFMI in young adulthood than those in the stable normal group (Additional file 1: Table S8). In contrast, participants in the decreasing—persistent low BMI group had lower levels of these indicators than the stable normal BMI group. Similar patterns were observed when males and females were analyzed separately (Additional file 1: Table S10).

**Table 1:** Characteristics of study participants according to body mass index trajectories

Characteristics	Increasing—persistent high (*n* = 74)	High—accelerated increasing (*n* = 209)	Increasing—accelerated resolving (*n* = 142)	Normal—above normal (*n* = 721)	Stable normal (*n* = 1608)	Decreasing—persistent low (*n* = 450)	*p*
Sex	*		***	***			< 0.001
Male	45 (60.8%)	95 (45.5%)	92 (64.8%)	407 (56.4%)	764 (47.5%)	202 (44.9%)	
Female	29 (39.2%)	114 (54.5%)	50 (35.2%)	314 (43.6%)	844 (52.5%)	248 (55.1%)	
Preterm birth	10 (13.5%)**	7 (3.3%)	26 (18.3%)***	48 (6.7%)	80 (5.0%)	11 (2.4%)*	< 0.001
Birth weight							
Kilogram	3.33 ± 0.63**	3.70 ± 0.46***	3.10 ± 0.76***	3.50 ± 0.56	3.53 ± 0.54	3.68 ± 0.51***	< 0.001
Z-scores	− 0.03 ± 1.40***	0.80 ± 0.91***	− 0.58 ± 1.73***	0.37 ± 1.17	0.46 ± 1.13	0.78 ± 1.00***	< 0.001
Cesarean section	15 (20.3%)*	29 (13.9%)	31 (21.8%)***	108 (15.0%)**	174 (10.8%)	52 (11.6%)	< 0.001
Exclusive breast feeding ≥ 4 months	59 (81.9%)	161 (78.9%)	103 (74.6%)	561 (79.6%)	1288 (81.5%)	375 (84.5%)	0.115
Current smoking at, yes							
24 years of age	11 (15.5%)	47 (24.5%)	20 (20.4%)	116 (19.4%)	276 (20.5%)	63 (16.9%)	0.319
26 years of age	1 (6.2%)	8 (14.8%)	2 (7.4%)	21 (10.1%)	68 (13.8%)	12 (7.9%)	0.327#
Parental education		***					0.013
Primary school/high school	35 (47.3%)	121 (57.9%)	65 (45.8%)	327 (45.4%)	707 (44.0%)	202 (44.9%)	
University	39 (52.7%)	88 (42.1%)	77 (54.2%)	393 (54.6%)	899 (56.0%)	248 (55.1%)	
Maternal characters during pregnancy							
Smoking during pregnancy	10 (13.7%)	40 (19.1%)***	22 (15.5%)	100 (13.9%)*	172 (10.7%)	45 (10.0%)	0.003
Age at delivery	30.83 ± 5.68	29.72 ± 4.67*	31.07 ± 4.70	30.67 ± 4.65	30.45 ± 4.39	30.47 ± 4.29	0.078
BMI at early pregnancy	26.51 ± 4.65***	25.10 ± 4.26***	24.32 ± 3.87***	23.42 ± 3.09***	22.41 ± 2.80	21.75 ± 2.73***	< 0.001
Diabetes mellitus	2 (2.8%)	0 (0.0%)	4 (2.9%)	13 (1.8%)	14 (0.9%)	7 (1.6%)	0.029#
Hypertension	5 (6.9%)*	6 (2.9%)	10 (7.2%)**	25 (3.5%)	42 (2.7%)	5 (1.1%)	0.002
Parity before the index person was born						*	0.011
One	17 (23.6%)	64 (31.1%)	40 (29.0%)	195 (27.5%)	479 (30.4%)	165 (37.3%)	
Two or more	11 (15.3%)	25 (12.1%)	8 (5.8%)	72 (10.2%)	134 (8.5%)	35 (7.9%)	

*BMI* Body mass index. The results were illustrated with mean ± standard deviation or numbers (proportions), respectively

^*^ *p* < 0.05

^**^ *p* < 0.01

^***^ *p* < 0.001 for comparisons between each trajectory group and the stable normal group

^#^Based on Fisher’s exact test

**Fig. 2:**
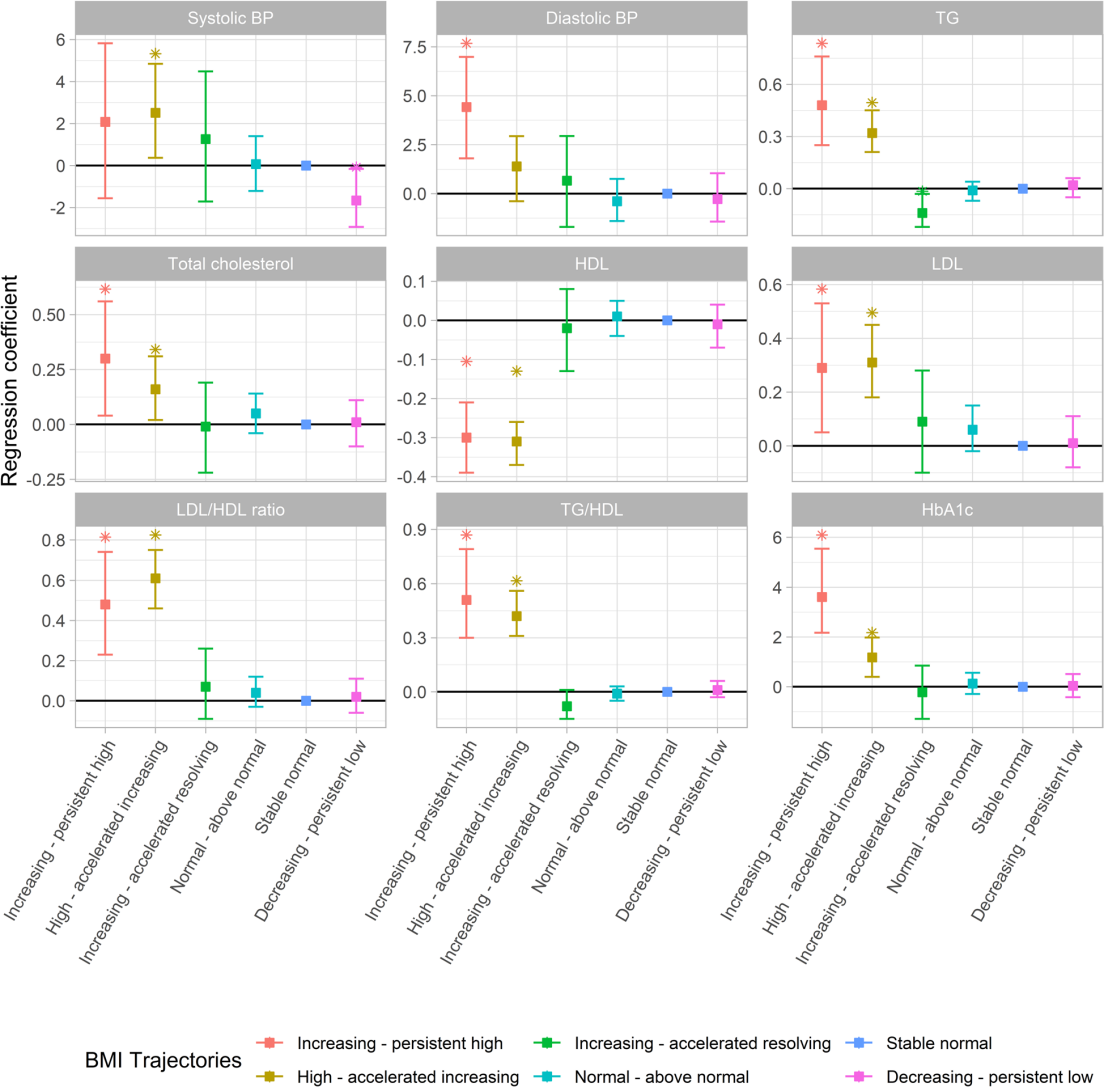
Association of BMI trajectories with blood pressure, blood lipids, and HbA1c at young adulthood determined by linear regression. The stable normal BMI group was the reference group. The y-axis displays the *β* coefficients along with their corresponding 95% confidence intervals. The models were adjusted for age, sex, smoking status, parental education, maternal smoking during pregnancy, maternal body mass index at early pregnancy, maternal hypertension, parity before the index person was born, and cesarean section. The statistics of this figure are presented in Table S11. HDL, high-density lipoprotein; LDL, low-density lipoprotein; HbA1c, hemoglobin A1C; TG, triglycerides. *: Significant difference between stable normal and other groups (*p* < 0.05)

Participants in the increasing—persistent high or high—accelerated increasing BMI groups had higher levels of total cholesterol [mean difference (MD) (95% confidence intervals (CI)): 0.30 (0.04–0.56) and 0.16 (0.02–0.31) mmol/L], TG [0.48 (0.25–0.76) and 0.32 (0.21–0.45) mmol/L], LDL [0.29 (0.05–0.53) and 0.31 (0.18–0.45) mmol/L], LDL/HDL ratio [0.48 (0.23–0.74) and 0.61 (0.46–0.75)], TG/HDL ratio [0.51 (0.30–0.79) and 0.42 (0.31–0.56)], and HbA1c [3.61 (2.17–5.54) and 1.18 (0.40–1.98) mmol/mol] but a lower level of HDL [− 0.30 (− 0.39 to − 0.21) and − 0.31 (− 0.37 to − 0.26) mmol/L] than those in the stable normal BMI group (Fig. 2 and Additional file 1: Table S11). Besides, there is decreased TG [− 0.14 (− 0.22, − 0.03) mmol/L] in the participants with increasing—accelerated resolving BMI group. There was no significant difference in these clinical chemistry measurements comparing the normal—above normal and the decreasing—persistent low BMI groups with the stable normal BMI group. Compared to the stable normal BMI group, the increasing—persistent high BMI group had higher diastolic blood pressure [4.42 (1.80–6.98) mmHg] and the high—accelerated increasing BMI group had higher systolic blood pressure [2.51 (0.37–4.84) mmHg]. In contrast, the decreasing—persistent low BMI group had lower systolic blood pressure. The results remained similar in males and females (Additional file 1: Tables S12 and S13). There was no substantial change in the associations observed for HDL and HbA1c in the increasing—persistent high BMI group and TG in the increasing—accelerated resolving BMI group, as well as for total cholesterol, HDL, LDL/HDL ratio, and TG/HDL ratio in the high—accelerated increasing BMI group, after accounting for the FMI in young adulthood, while most other associations shifted towards the null (Additional file 2: Fig. S5). Besides, similar trend changes were observed in models that accounted for BMI z-scores at 24 years (Additional file 1: Table S14), while additionally adjusting for BMI z-scores at birth did not change the associations (Additional file 1: Table S15). Furthermore, the increasing—persistent high and high—accelerated increasing groups also exhibited heightened clinical-related cardiometabolic risks (odds ratio = 4.45 (2.17–9.14) and 3.42 (2.18–5.38), respectively, Additional file 2: Fig. S6).

**Fig. 3:**
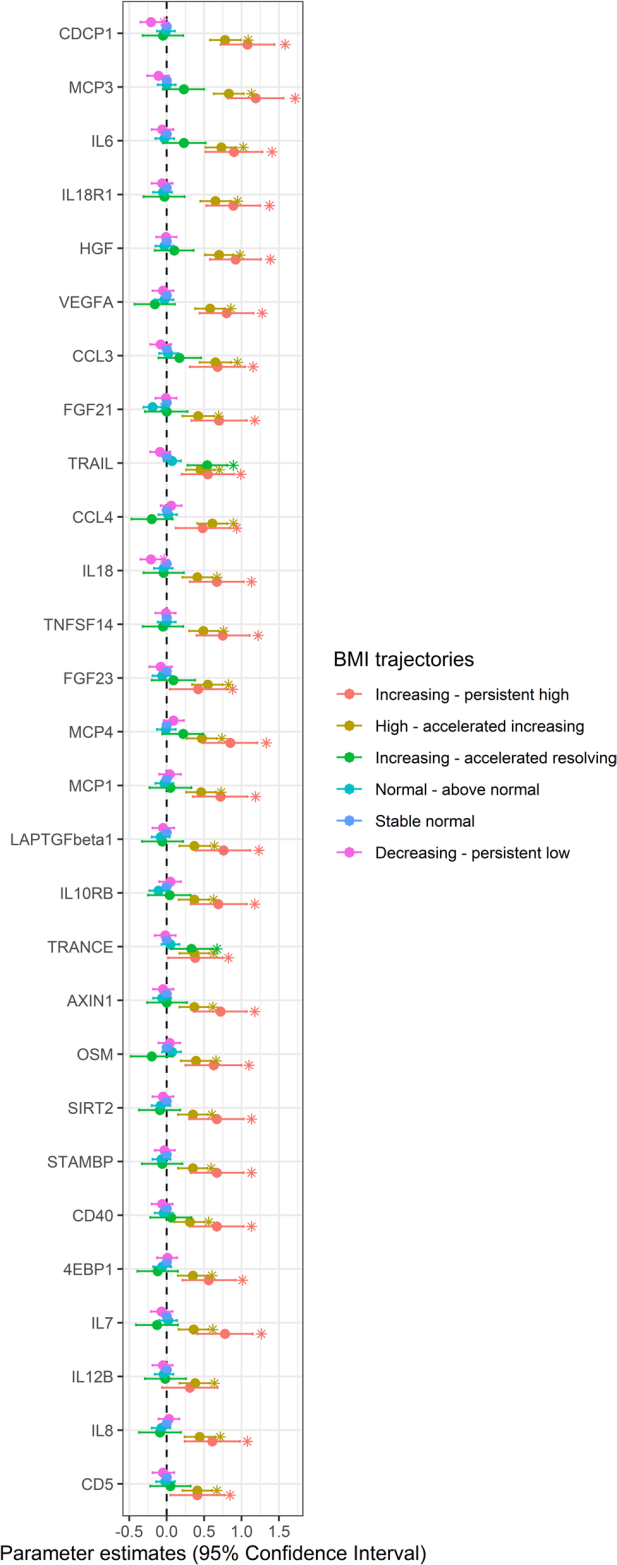
Association of BMI trajectories with circulating inflammatory proteins determined by linear regression. The stable normal BMI group was the reference group. The x-axis displays the *β* coefficients along with their corresponding 95% confidence intervals. The models were adjusted for sex, smoking status, parental education, maternal smoking during pregnancy, maternal BMI at early pregnancy, maternal hypertension, parity before the index person was born, and cesarean section. The statistics of this figure are presented in Table S17. *: Significant difference between stable normal and other groups (*p* < 0.05)

Participants in the increasing—persistent high and high—accelerated increasing BMI groups had a higher leukocyte cell count in young adulthood than those in the stable normal BMI group (Additional file 1: Table S16). These two groups also had higher levels of 28 inflammation-related proteins than the stable normal BMI group (Fig. 3 and Additional file 1: Table S17). Besides, several of these proteins exhibited high or moderate expression levels in the heart muscle, smooth muscle, and adipose tissue (Additional file 2: Fig. S7).

## Discussion

In our longitudinal birth cohort study, we identified six distinct BMI trajectories from birth until young adulthood, namely the increasing—persistent high, high—accelerated increasing, increasing—accelerated resolving, normal—above normal, stable normal, and decreasing—persistent low BMI group. We found that the increasing—persistent high and the high—accelerated increasing BMI groups had markedly higher levels of several blood lipids (total cholesterol, TG, LDL, LDL/HDL ratio, TG/HDL ratio) and HbA1c but a lower level of HDL than the stable normal BMI group. The associations for HDL, TG/HDL ratio, and HbA1c were still observed after adjusting for FMI in young adulthood. Both the increasing—persistent high and high—accelerated increasing BMI groups had higher leukocyte cell count and higher levels of a range of inflammation-related proteins. There was no significant difference in the clinical cardiometabolic profiles in young adulthood between the increasing—accelerated resolving group and the stable normal BMI group.

### Comparison with previous studies

Previous studies showed diverse BMI trajectories from birth or young age until later life. In our literature review (Table 2 and Additional file 1: Table S18), we included studies that (1) applied latent class group modeling or equivalents to identify BMI trajectories from childhood, (2) classified study individuals by BMI trajectories, and analyzed the associations with cardiovascular risk in later life, and (3) outcomes were measured no later than middle age (40–50 years), leading to a total of 31 eligible studies.

**Table 2:** Summary of previous studies on the association of BMI trajectories in childhood with cardiovascular risk in young adulthood

Study; country	Sample size	BMI assessment points	Measurement time: outcomes	Summary of the results	
				**BMI trajectories**	**Conclusions**
**Birth cohort**					
[10] South Korea	249	Seven times: at birth, 3, 5, 7, 8, 9, 10, 11, 12 years	Age 13–15 years: hs-CRP, IL-6, and CMR score	Three trajectories: low-stable, moderate-stable, and high-stable increasing BMI	• Compared to the group with low-stable increasing BMI, the other two groups had higher levels of hs-CRP, IL-6, and CMR score
[30] USA	322	Six times: at birth, 2, 3, 4.5, 6, and 7.5 years	Age 7.5 years: waist circumference, SBP, DBP, HbA1c, HDL, and TC	Three trajectories: low-stable, high-stable, and increasing BMI	• Compared to the group with low-stable BMI, those with high-stable or increasing BMI had higher SBP and waist circumference but lower levels of HDL • No difference was observed for other outcomes
[31] Spain	489	Seven times: at birth, 0.5, 1, 2, 4, 7, and 9 years	Age 11 years: SBP, DBP, PWV, and microvascular function (central retinal arteriolar/venular equivalent)	Five trajectories: high birth size and accelerated gain, high birth size and slow gain, low birth size and accelerated gain, average birth size and slow gain, and low birth size and slow gain	• Compared to children with average birth size and slow BMI gain, those with low birth size and accelerated BMI gain had higher SBP, DBP, and PWV, and those with high birth size and accelerated BMI gain had higher SBP
[12] Guatemala	856	3–22 times from age 1 month to 42 years	Age 37–54 years: elevated triglycerides (≥ 150 mg/dL), low HDL (< 50 mg/dL for women and < 40 mg/dL for men), diabetes (plasma glucose ≥ 126 mg/dL, post-challenge glucose ≥ 200 mg/dL, or use of diabetes medication), hypertension, and metabolic syndrome^a^	For men, two trajectories: low and medium/high BMI For women, two trajectories: low and high BMI	• In men, the trajectory of medium/high BMI had higher odds of elevated triglycerides and low HDL than the trajectory of low BMI. No observation was observed for other outcomes • In women, no difference in the studied outcomes was observed between the trajectories of high and low BMI
[32] Australia	1288	Six times: every 2 years from age 0–1 to 11–12 years	Age 11–12 years: retinal vascular caliber	Five trajectories: low, average, always high, always very high, and low to high BMI	• Compared to the trajectory of average BMI, the trajectory of always very high BMI had narrower retinal vascular caliber • No difference was found for other trajectories compared to the trajectory of average BMI
[33] Australia	190	Multiple times from birth to 14 years	Age 14 years: waist circumference, TC, HDLC, LDL, triglycerides, apolipoprotein A1, apolipoprotein B, Apo A1/B, SBP, DBP, cIMT, and PWV	Three trajectories: normal, early-rising, and late-rising BMI	• Compared to the trajectory of normal BMI, both trajectories of early-rising and late-rising BMI had higher levels of Apo B, Apo A1/B, and SBP and a lower level of Apo A1 while the level of HDL was higher in late-rising and lower in early-rising BMI trajectory
[34] Australia	1197	Eight times: at birth, 1, 2, 3, 5, 8, 10, and 14 years	Age 14 years: insulin and homeostasis model assessment of insulin resistance	Seven trajectories: very low stable, moderately low stable, optimal normal growth, rising to moderate, falling to moderate, rising to high, and stable high BMI	• In boys, compared to the trajectory of optimal normal growth, the trajectories of stable high, rising to high, and rising to moderate BMI had higher levels of insulin and insulin resistance, while the trajectory of very low stable BMI had lower levels of insulin and insulin resistance • In girls, compared to the trajectory of optimal normal growth, the trajectories of stable high and rising to high BMI had higher levels of insulin and insulin resistance
[35] Canada	1166	At least one time: up to twice a year from 0 to 2 years and every year until age 5 years	Age 3–5 years: cardiometabolic risk^b^	Four trajectories: catch-up, stable high, stable low, and rapid accelerating BMI	• Compared to children with stable low BMI, those with the trajectories of catch-up, stable high, and rapid accelerating BMI had a higher cardiometabolic risk score
[36] Ethiopia	453	Median nine times: at birth, 1.5, 2.5, 3.5, 4.5, 6, and 12 months, 1.5, 2, 3, 4, and 5 years	Age 5 years: glucose, HbA1c, insulin, C-peptide, HOMA-IR, TC, LDL, HDL, triglyceride, SBP, DBP, waist circumference, fat mass, and fat-free mass	Four trajectories: stable low, normal, rapid catch-up to high, and slow catch-up to high BMI	• Children with stable low BMI had lower LDL, HDL, waist circumference, fat mass, and fat-free mass than those with normal BMI • Children with rapid catch-up to high BMI had higher C-peptide, triglyceride, waist circumference, fat mass, and fat-free mass than those with normal BMI • Children with slow catch-up to high BMI had higher fat mass than those with normal BMI
[11] Singapore	1170	Eight times: at birth, 3, 6, 9, 12, 15 and 18 months, and 2 years	Age 5 years: waist-to-height ratio, the sum of skinfolds, fat-mass index, lean-mass index, SBP, and DBP	Four trajectories: stable low, normal, and stable high BMI, and rapid BMI gain after 3 months	• Children with stable low BMI had lower waist-to-height ratio, sum of skinfolds, fat-mass index, lean-mass index, SBP, and DBP than those with normal BMI • Children with stable high BMI had higher waist-to-height ratio, sum of skinfolds, and lean-mass index than those with normal BMI • Children with rapid BMI gain after 3 months had higher waist-to-height ratio, sum of skinfolds, and fat-mass index than those with normal BMI
[9]; Australia	187	11–16 times: at birth, 2 weeks, 2, 4, 8, 12, 18, and 24 months, 6 times between 4 and 6.5 years, as well as 10 and 14 years	Age 14 years: SBP, DBP, augmentation index, PWV, carotid intima-media thickness, and retinal arteriole-to-venule ratio	Three trajectories: consistently overweight, high normal, and low normal BMI	• Children with consistently overweight had higher augmentation index than those with low normal BMI in early childhood • No associations were observed for any other outcomes
**Others**					
[37] USA	338	Six times during ages 2–11 years	Age 7–11 years: CRP, leptin, adiponectin, SBP, DBP, HbA1C, TC, HDL, LDL, triglycerides, triglyceride/HDL ratio, fasting glucose, fasting insulin, oxLDL, PWV, CMR scores	Two trajectories: moderate-decrease and marked-increase BMI	• Compared to children with moderate-decrease BMI, those with marked-increase BMI had higher levels of CRP, leptin, triglycerides, triglyceride/HDL ratio, HbA1C, fasting glucose and insulin, SBP, DBP, and overall CMR score, as well as lower levels of adiponectin and HDL
[17] China	2167	At least three times during age 6–48 years	Age 36–48 years: PWV and cIMT	Three trajectories: low-, moderate-, and high-increasing BMI	• Compared to the trajectory of low-increasing BMI, both trajectories of moderate- and high-increasing BMI had higher levels of PWV and cIMT
[15] Denmark	13,438	At two times during ages 6–15 years	Age 30–70 years: type 2 diabetes and coronary heart disease	Five parallel trajectories with consistently increasing BMI	• Individuals with trajectories of higher BMI had a higher risk of type 2 diabetes than those with the trajectory of the lowest BMI during childhood • The association was not observed for coronary heart disease
[38] China	24,426	At least four times during age 6–17 years	Age 12–17 years: hypertension	Four trajectories: low-, medium-, high-, and highest-increasing BMI	• Compared to the trajectory of medium-increasing BMI, the trajectory of low-increasing BMI had a lower odd of hypertension while the trajectories of high- and highest-increasing BMI had a higher odd of hypertension
[8] China	1825	At least three times during age 6–48 years	Age 36–48 years: albuminuria (urinary albumin-to-creatinine ratio ≥ 30 mg/g)	Three trajectories: low-, moderate-, and high-increasing BMI	• The trajectory of high- but not moderate-increasing BMI had a higher odd of albuminuria than the low-increasing BMI trajectory
[39] China	17,816	At least eight times during age 7–18 years	Age 16–18 years: elevated blood pressure (SBP ≥ 130 mmHg or DBP ≥ 80 mmHg)	Four trajectories: constant low, high-decreasing, low-rising, and constant high BMI	• Compared to the trajectory of constant low BMI, a higher risk of elevated blood pressure was observed in the other trajectories among either boys or girls
[40] Australia	1312	Four times: at ages 14, 17, 20, and 22 years	Age 14, 17, 20, and 22 years: CRP	Four trajectories: low, medium, medium-increasing, and high-increasing BMI	• Individuals in the high-increasing BMI trajectory were more likely to be in the group of stable-high CRP than other BMI trajectories
[41] China	1907	Three to six times from age 5–19 years	Age 14–22 years: hypertension	Three trajectories: normal increasing, resolving, and high increasing BMI	• The trajectory of high increasing BMI had a higher risk of hypertension than that of normal increasing BMI • No difference was observed between trajectories of normal increasing and resolving BMI
[42] UK	3549	Ten times at ages 7, 8, 9, 10, 11, 12, 13, 15, 17, and 24 years	Age 24 years: SBP, DBP, TC, HDL, LDL, triglycerides, insulin, glucose, CRP, cIMT, PWV, fat mass, trunk fat mass, lean fat, trunk lean fat, peripheral lean fat, relative wall thickness, left ventricular mass indexed to height in m^3^, peak mitral annular velocity in systole, early/late mitral inflow velocity (E/A ratio), and early mitral inflow velocity/mitral annular early diastolic velocity (E/e′ ratio)	Six trajectories: normal weight (nonlinear), normal weight (linear), normal weight increasing to overweight, normal weight or overweight, normal weight increasing to obesity, and overweight or obesity	• Compared to the trajectory of normal-weight (nonlinear), the trajectory of normal-weight (linear) had more trunk fat, higher blood pressures, higher left ventricular mass indexed to height in m^3^, and insulin. The differences were more pronounced for the trajectories of normal-weight-or-overweight and normal-weight-increasing-to-overweight • Compared to the trajectory of normal-weight (nonlinear), the trajectories of overweight-or-obesity and normal-weight-increasing-to-obesity had worse levels of almost all outcomes
[43] China	1872	At least two times during age 6–18 years	Age 18–37 years: hypertension	Five trajectories: low slow-increasing, low moderate-increasing, low rapid-increasing, moderate-increasing, and elevated-decreasing BMI	• Compared with the trajectory of low slow-increasing BMI, the groups with low moderate-increasing, low rapid-increasing, and moderate-increasing BMI had a higher risk of hypertension
[44] Taiwan	1387	At least two times during the age 13–18 years	Early adulthood (mean 33 years): diabetes	Five trajectories: stable weight, rapid decrease then stable, persistent decrease, slow decrease, and increased then stable	• No significant difference in the risk of diabetes was observed across the five BMI trajectories
[14] Denmark	2466	At least 3 times during age 6–14 years	Mean age 40 years: waist circumference, SBP, DBP, triglycerides, HDL, LDL, total cholesterol, remnant cholesterol, lipoprotein(a), apolipoprotein B, HbA1c, and self-reported diabetes	Five parallel trajectories with consistently increasing BMI during childhood	• Compared to the trajectory with the lowest BMI throughout childhood, the risks of waist circumference and self-reported diabetes were higher in the top three trajectories with higher BMI but were not different from that in the trajectory with the second lowest BMI. Such a pattern was not observed in other cardiovascular risk factors
[45] Australia	1811	Five times: every 2 years between ages 2 to 3 and 10 to 11 years	Age 11 to 12 years: CMR scores, carotid-femoral pulse wave velocity, and carotid intima-media thickness	Five trajectories: low, healthy, low to high, high, and always very high BMI	• Compared to the trajectory of low BMI, the trajectories of low to high, high, and always very high BMI had higher levels of CMR scores, carotid-femoral pulse wave velocity, and carotid intima-media thickness
[46] China	9286	At least eight times during age 8–18 years	Age 16–18 years: hypertension	Four trajectories: low, middle, high, and very high increasing BMI	• Compared to children with middle increasing BMI, those with low increasing BMI had a lower risk of hypertension, whereas those with high or very high increasing BMI had a higher risk of hypertension
[47] China	2839	At 3 times during ages 6–48 years	Age 18–48 years: type 2 diabetes, hypertension, high-risk lipid levels, high-risk cIMT, high-risk baPWV, and left ventricular hypertrophy	Three trajectories: low-, moderate-, and high-increasing BMI (trajectories of low- and moderate-increasing BMI were parallel)	• Both moderate- and high-increasing groups had a higher risk of hypertension, type 2 diabetes, high-risk total cholesterol, and high-risk HDL
[48] Germany	689	At least three times during age 4–18 years	Age 18–39 years: SBP, DBP, triglyceride, HDL, FPG, CRP, IL‐6, IL‐18, adiponectin, chemerin, and leptin	For girls, three trajectories: low-, middle-, and high-normal weight For boys, four trajectories: low-, middle-, and high-normal weight, and overweight	• BMI trajectories were associated with HDL and IL-18 in males and with DBP and IL-6 in females
[16] Finland	2631	Mean five times during age 6–49 years	Age 34–49 years: type 2 diabetes, high-risk lipid levels, hypertension, and high cIMT	Six trajectories: stable normal, resolving, progressively overweight, progressively obese, rapidly overweight/obese, and persistent increasing overweight/obese	• The trajectories with worsening or persisting obesity had a higher risk of type 2 diabetes, hypertension, high-risk lipid, and high cIMT in adulthood compared to the trajectory with consistently normal BMI
[49] USA	626	3–12 times during age 5–18 years	Mean age 24 years: intima-media thickness and left ventricular mass index	Three trajectories: normal, moderate-increasing, and high-increasing BMI	• Compared to the group of normal BMI trajectory, those with moderate- and high-increasing BMI had higher intima-media thickness and left ventricular mass index
[50] Portugal	1763	Three times: age 13, 17, and 21 years	Age 21 years: SBP, DBP, insulin resistance, triglycerides, HDL, and LDL	Three trajectories: normal, high-declining, and high-increasing BMI	• Compared to the trajectory of normal BMI, the trajectory of high-increasing BMI had higher levels of SBP, DBP, insulin resistance, triglycerides, and LDL but a lower level of HDL • The trajectory of high-declining BMI had a lower level of HDL than the trajectory of normal BMI
[51] South Africa	1824	At least two times during age 5–18 years	Age 17–18 years: SBP, DBP, MAP, and elevated blood pressure (for those under 18 years old: SBP or DBP equal or over 90th percentile of the study individuals; for those at or over 18 years old: prehypertension [SBP ≥ 120 mmHg or DBP ≥ 80 mmHg] or hypertension)	For girls, four trajectories: normal weight, late onset overweight, early onset obese to overweight, and early onset obese to morbidity obese For boys, three trajectories: normal weight, early onset overweight to normal weight, and early onset overweight to obese	• In girls, compared to the trajectory of normal weight, the trajectories of early onset obese to overweight and early onset obese to morbidity obese had higher levels of SBP, DBP, and MAP, as well as a higher odd of elevated blood pressure • In boys, compared to the trajectory of normal weight, the trajectory of early onset overweight to obese had higher levels of SBP, DBP, and MAP

*baPWV* brachial-ankle pulse wave velocity, *BMI* Body mass index, *cIMT* carotid intima-media thickness, *CMR* metabolic syndrome risk, *DBP* Diastolic blood pressure, *FPG* Fasting plasma glucose, *HbA1c* Hemoglobin A1c, *HDL* High-density lipoprotein, *HOMA-IR* Homeostasis model assessment of insulin resistance, insulin (μU/mL) × glucose (mmol/L)/22.5, *hs-CRP* High-sensitivity C-reactive protein, *LDL* Low-density lipoprotein, *MAP* Mean arterial pressure, *oxLDL* oxidized low-density lipoprotein, *PWV* Pulse wave velocity, *SBP* Systolic blood pressure, *TC* Total cholesterol

^a^Hypertension was defined as systolic blood pressure ≥ 130 mmHg and/or diastolic blood pressure ≥ 80 mmHg and/or anti-hypertensive medication use. Metabolic syndrome was defined if people had at least three following conditions: (1) abdominal obesity (waist circumference > 88 cm for women; > 102 cm for men); (2) fasting glucose ≥ 100 mg/dL or diabetes medication use; (3) triglycerides ≥ 150 mg/dL or statin use; (4) high density lipoprotein < 40 mg/dL in men or < 50 mg/dL in women; and (5) systolic blood pressure ≥ 130 mmHg, diastolic blood pressure ≥ 85 mmHg, and/or hypertension medication use

^b^Cardiometabolic risk was defined as the sum of age- and sex-standardized waist circumference, systolic blood pressure, blood tests for glucose, log-triglycerides, and the negative of high-density lipoprotein, divided by the square root of 5

Three trajectories, i.e., groups of stable BMI within the normal range, persistently high BMI, and persistently low BMI over time, are generally observed. It is suggested by some [13, 15, 16, 39], though not all [9, 14], of them that compared to the group of stable BMI within the normal range over time, the persistently high BMI group had higher risks of hypertension and diabetes, higher levels of systolic blood pressure (SBP), diastolic blood pressure (DBP), HbA1c, glucose, insulin, insulin resistance, total cholesterol, TG, LDL, and ApoB, as well as a lower level of HDL. In contrast, the persistently low BMI group had a lower risk of hypertension and lower levels of SBP, DBP, LDL, and insulin [11, 13]. These findings are predominantly observed in studies with follow-up from early adulthood up to middle age, while studies with follow-up limited to adolescence, particularly those birth cohort studies included in our literature review, struggle to replicate those findings. Cardiometabolic risks induced by overweight/obesity may be established at an early age, but they are generally unlikely to be detected before adulthood. The present study, with follow-up extending until young adulthood, yielded findings consistent with prior studies focusing on BMI trajectories from early to middle adulthood. Additionally, we found that the high—accelerated increasing BMI group had higher levels of total cholesterol, triglycerides, LDL, HDL/LDL ratio, HbA1c, and TG/HDL ratio, and a lower level of HDL than the stable normal BMI group.

The role of long-term high BMI may present differently in glucose and lipid mechanisms. We observed that differences in TG/HDL ratio, a surrogate marker of insulin resistance [52], comparing the increasing—persistent high BMI group and the high—accelerated increasing BMI group to the stable normal BMI group remained unchanged after adjusting for FMI measured at young adulthood. However, no increased cardiometabolic markers were observed in the increasing—accelerated resolving BMI group. Similarly, Buscot and colleagues reported that individuals who became overweight/obese during young adulthood but later resolved their BMI had a lower risk of diabetes than those who remained consistently obese/overweight [16]. This suggests that adverse changes in glucose metabolisms may commence with the onset of overweight or obesity and are likely to be mitigated by the normalization of high BMI at an earlier age. In contrast, lipids are likely to reflect current body fat status. We found that the elevated blood lipid levels in the increasing—persistent high and high—accelerated increasing BMI groups compared to the stable normal BMI group diminished after adjusting for the FMI in young adulthood. A previous study reported similar patterns in men specifically that the associations of BMI trajectories with TG and HDL approached the null after adjustment for body adiposity at the time of outcome evaluation [12]. However, current BMI is to a large extent dependent on earlier BMI. Thus, obesity prevention targeting children may have substantial significance in reducing cardiometabolic risk in later life.

Excess fat mass is likely to induce systemic inflammation [3, 53]. Our previous work suggested that a high percentage of body fat was associated with increased levels of inflammation-related plasma proteins [54]. The present study corroborates our earlier findings. Notably, similar findings regarding increased protein levels were reported in the UK Biobank [55]. Furthermore, a higher level of leukocytes was observed in the two groups in comparison to the stable normal BMI group. Although only a few studies of BMI trajectories have focused on inflammatory biomarkers, their findings are largely similar. Both Kim et al. and Oluwagbemigun et al. reported that individuals with high BMI had a higher level of IL-6 than those with low BMI [10, 48]. Similar associations were observed for CRP in two prior studies [10, 37].

This study had several strengths. First, our population-based longitudinal cohort study design and large sample size make our findings important from a public health point of view. Second, the analysis of cardiometabolic profiles concerning BMI trajectories is particularly advantageous in young adulthood when it is not too early to distinguish the risk across BMI trajectories and not too late to be able to reduce cardiometabolic risk induced by modifiable factors [56]. Several limitations, however, should be noted in this study. First, not all participants in BAMSE were included in the analysis due to the lack of information on BMI and outcomes. The participants included in the BMI trajectory analysis had higher socioeconomic status and lower smoking exposure compared to those excluded, which may introduce potential selection bias. Second, the generalizability of our findings may be limited to the context of a similar welfare system and lifestyle as in Sweden. Third, only a subset of participants had body fat mass index data at age 24. Therefore, our findings on the potential influence of current body fat status on the associations between BMI trajectories and cardiometabolic profiles require further replication.

## Conclusions

Participants with normal birth weight, increasing rapidly during childhood and remaining high in adolescence, or with high birth weight and increasing over time have higher risks of adverse cardiometabolic profiles (i.e., lipids, glucose metabolism, and inflammation) than those with stable normal BMI. However, a raised BMI in childhood may not be inherently harmful to cardiometabolic health, provided it does not persist into adulthood. Obesity prevention targeting children may reduce the risk of adverse cardiometabolic profiles and subsequently lower the risk of cardiometabolic diseases in later life.

## Supplementary Information

Additional file 1: Tables S1–S18. Table S1 The data source of body mass index used in the current study. Table S2 Full names of the 92 proteins included in the Proseek Multiplex Inflammation I panel. Table S3 Inflammation-related protein levels at 24 years according to body mass index trajectories. Table S4 Characteristics of the included and excluded participants. Table S5 The absolute error loss across latent class mixture models incorporating linear, quadratic, and cubic terms. Table S6 The results of latent class mixture models incorporating quadratic terms. Table S7 The mean and standard deviation of BMI and BMI z-scores for all the follow-up points for the BMI groups. Table S8 Characteristics of males in each body mass index trajectory. Table S9 Characteristics of females according to body mass index trajectories. Table S10 Bioimpedance at 24 years and 26 years according to body mass index trajectories. Table S11 Mean differences and 95% confidence intervals for the associations of body mass index trajectories with cardiometabolic profile at late adolescence and young adulthood by linear regression. Table S12 Mean differences and 95% confidence intervals for the associations of body mass index trajectories with cardiometabolic profile at late adolescence and young adulthood by linear regression in males. Table S13 Mean differences and 95% confidence intervals for the associations of body mass index trajectories with cardiometabolic profile at late adolescence and young adulthood by linear regression in females. Table S14 Mean differences and 95% confidence intervals of the sensitivity analysis which additionally included the BMI z-scores at 24 years. Table S15 Mean differences and 95% confidence intervals of the sensitivity analysis which additionally included the BMI z-scores at birth. Table S16 Blood cell counts at 24 years according to body mass index trajectories. Table S17 Mean differences and 95% confidence intervals for the associations of body mass index trajectories with inflammation-related proteins in young adulthood by linear regression. Table S18 The list of studies included in the literature review.

Additional file 2: Figures S1–S7. Fig. S1 Flow chart of study participants in the BAMSE cohort. Fig. S2 Body mass index trajectories from birth to young adulthood in the BAMSE cohort. Fig. S3 Body mass index z-scores for participants with or without BMI data at 24 years of age in six BMI groups. Fig. S4 Body mass index values for participants with or without BMI data at 24 years of age in six BMI groups. Fig. S5 Association of body mass index trajectories with blood pressure, blood lipid, and HbA1c in young adulthood after additionally adjusting for fat mass index determined by linear regression. Fig. S6 Association of BMI trajectories with any heightened cardiometabolic risk at young adulthood determined by logistic regression. Fig. S7 Protein expression levels in the heart muscle, smooth muscle, and adipose tissue.

Additional file 3. Additional methods.

## Data Availability

The data that support the findings of this study are available on reasonable request from the principal investigators of the BAMSE cohort (I.K., A.B., and E.M.). The data are not publicly available due to the privacy and confidentiality of the research participants.

## Abbreviations

BAMSE: Child [Barn], Allergy, Milieu, Stockholm, Epidemiological
BMI: Body mass index
BIC: The Bayesian Information Criterion
CI: Confidence intervals
CVD: Cardiovascular diseases
DBP: Diastolic blood pressure
EDTA: Ethylenediaminetetraacetic acid
FFMI: Fat-free mass index
FMI: Fat mass index
HbA1c: Hemoglobin A1C
HDL: High-density lipoprotein
LCMM: Latent class mixture modeling
LDL: Low-density lipoprotein
MD: Mean difference
SBP: Systolic blood pressure
TG: Triglyceride
