# Supplementary material for: BMI trajectories from birth to young adulthood associate with distinct cardiometabolic profiles
Source: BMC Med. 2024 Nov 5;22:510. doi: 10.1186/s12916-024-03741-0 (PMC11539615; doi:10.1186/s12916-024-03741-0)
Supplement: Supplementary file 1 — Additional file 1: Tables S1–S18. Table S1 The data source of body mass index used in the current study. Table S2 Full names of the 92 proteins included in the Proseek Multiplex Inflammation I panel. Table S3 Inflammation-related protein levels at 24 years according to body mass index trajectories. Table S4 Characteristics of the included and excluded participants. Table S5 The absolute error loss across latent class mixture models incorporating linear, quadratic, and cubic terms. Table S6 The results of latent class mixture models incorporating quadratic terms. Table S7 The mean and standard deviation of BMI and BMI z-scores for all the follow-up points for the BMI groups. Table S8 Characteristics of males in each body mass index trajectory. Table S9 Characteristics of females according to body mass index trajectories. Table S10 Bioimpedance at 24 years and 26 years according to body mass index trajectories. Table S11 Mean differences and 95% confidence intervals for the associations of body mass index trajectories with cardiometabolic profile at late adolescence and young adulthood by linear regression. Table S12 Mean differences and 95% confidence intervals for the associations of body mass index trajectories with cardiometabolic profile at late adolescence and young adulthood by linear regression in males. Table S13 Mean differences and 95% confidence intervals for the associations of body mass index trajectories with cardiometabolic profile at late adolescence and young adulthood by linear regression in females. Table S14 Mean differences and 95% confidence intervals of the sensitivity analysis which additionally included the BMI z-scores at 24 years. Table S15 Mean differences and 95% confidence intervals of the sensitivity analysis which additionally included the BMI z-scores at birth. Table S16 Blood cell counts at 24 years according to body mass index trajectories. Table S17 Mean differences and 95% confidence intervals for the associations of body mass ind [file 12916_2024_3741_MOESM1_ESM.pdf]

28 October 2024

Additional file 1

“BMI trajectories from birth to young adulthood associate with distinct cardiometabolic profiles”

Authors:

Gang Wang*#; Dang Wei#; Simon Kebede Merid; Sandra Ekström; Susanna Klevebro; Natalia Hernandez-Pacheco; Sophia Björkander; Petter Ljungman; Inger Kull; Jochen M Schwenk; Anna Bergström##; Erik Melén##

# Equal contributors of first authors.

Equal contributors of last authors.

* Corresponding Author

Table S1. The data source of body mass index used in the current study.

Age, years	0	0.5	1	1.5	2	3	4	5	7	8	10	12	16	24
Swedish Medical Birth Register	X N=3998													
Clinical investigations							X N=2937			X N=2620			X N=2605	X N=2270
School and healthcare records		X N=2317	X N=2302	X N=2254	X N=1601	X N=1296	X N=2268	X N=2210	X N=2473		X N=2242	X N=2255		
Self-reported	X N=4044											X N=2715	X N=3058	X N=2991

"X" indicates data were available in that data source. N: number.

Table S2. Full names of the 92 proteins included in the Proseek Multiplex Inflammation I panel.

Proteins	Full names	Uniprot ID
4EBP1	Eukaryotic translation initiation factor 4E-binding protein 1	Q13541
ADA	Adenosine Deaminase	P00813
ARTN	Artemin	Q5T4W7
AXIN1	Axin-1	O15169
BetaNGF	Beta-nerve growth factor	P01138
CASP8	Caspase-8	Q14790
CCL3	C-C motif chemokine 3	P10147
CCL4	C-C motif chemokine 4	P13236
CCL11	C-C motif chemokine 11	P51671
CCL19	C-C motif chemokine 19	Q99731
CCL20	C-C motif chemokine 20	P78556
CCL23	C-C motif chemokine 23	P55773
CCL25	C-C motif chemokine 25	O15444
CCL28	C-C motif chemokine 28	Q9NRJ3
CD5	Cluster of differentiation 5	P06127
CD6	Cluster of differentiation 6	P30203
CD8A	Cluster of differentiation 8a	P01732
CD40	Cluster of differentiation 40	P25942
CD244	Natural Killer cell receptor 2B4	Q9BZW8
CDCP1	CUB domain-containing protein 1	Q9H5V8
CSF1	Macrophage colony-stimulating factor 1	P09603
CST5	Cystatin D	P28325
CX3CL1	Fractalkine	P78423
CXCL1	C-X-C motif chemokine 1	P09341
CXCL5	C-X-C motif chemokine 5	P42830
CXCL6	C-X-C motif chemokine 6	P80162
CXCL9	C-X-C motif chemokine 9	Q07325
CXCL10	C-X-C motif chemokine 10	P02778
CXCL11	C-X-C motif chemokine 11	O14625
DNER	Delta and Notch-like epidermal or growth factor-related receptor	Q8NFT8
ENRAGE	Protein S100-A12	P80511

FGF5	Fibroblast growth factor 5	P12034
FGF19	Fibroblast growth factor 19	O95750
FGF21	Fibroblast growth factor 21	Q9NSA1
FGF23	Fibroblast growth factor 23	Q9GZV9
Flt3L	Fms-related tyrosine kinase 3 ligand	P49771
GDNF	Glial cell line-derived neurotrophic factor	P39905
HGF	Hepatocyte growth factor	P14210
IFNgamma	Interferon-gamma	P01579
IL1 alpha	Interleukin-1 alpha	P01583
IL2	Interleukin-2	P60568
IL2RB	Interleukin-2 receptor subunit beta	P14784
IL4	Interleukin-4	P05112
IL5	nterleukin-5	P05113
IL6	Interleukin-6	P05231
IL7	Interleukin-7	P13232
IL8	Interleukin-8	P10145
IL10	Interleukin-10	P22301
IL10RA	Interleukin-10 receptor subunit alpha	Q13651
IL10RB	Interleukin-10 receptor beta	Q08334
IL12B	Interleukin-12 subunit beta	P29460
IL13	Interleukin-13	P35225
IL15RA	Interleukin-15 receptor alpha	Q13261
IL17A	Interleukin-17A	Q16552
IL17C	Interleukin-17C	Q9P0M4
IL18	Interleukin-18	Q14116
IL18R1	Interleukin-18 receptor 1	Q13478
IL20	nterleukin-20	Q9NYY1
IL20RA	Interleukin-20 receptor subunit alpha	Q9UHF4
IL22 RA1	Interleukin-22 receptor subunit alpha-1	Q8N6P7
IL24	Interleukin-24	Q13007
IL33	Interleukin-33	O95760
LAPTGFbeta1	Latency-associated peptide transforming or growth factor beta-1	P01137
LIF	Leukemia inhibitory factor	P15018

LIFR	Leukemia inhibitory factor receptor	P42702
MCP1	Monocyte chemotactic protein 1	P13500
MCP2	Monocyte chemotactic protein 2	P80075
MCP3	Monocyte chemotactic protein 3	P80098
MCP4	Monocyte chemotactic protein 4	Q99616
MMP1	Matrix metalloproteinase-1	P03956
MMP10	Matrix metalloproteinase-10	P09238
NRTN	Neurturin	Q99748
NT3	Neurotrophin-3	P20783
OPG	Osteoprotegerin	O00300
OSM	Oncostatin-M	P13725
PDL1	Programmed cell death ligand 1	Q9NZQ7
SCF	Stem Cell Factor	P21583
SIRT2	SIR2-like protein 2	Q8IXJ6
SLAMF1	Signaling lymphocytic activation molecule	Q13291
ST1A1	Sulfotransferase 1A1	P50225
STAMBP	STAM-binding protein	O95630
TGFalpha	Transforming Growth Factor alpha	P01135
TNF	Tumor necrosis factor	P01375
TNFB	Tumor necrosis factor beta	P01374
TNFRSF9	Tumor necrosis factor receptor superfamily member 9	Q07011
TNFSF14	Tumor necrosis factor ligand superfamily member 14	O43557
TRAIL	TNF-related apoptosis-inducing ligand	P50591
TRANCE	TNF-related activation-induced cytokine	O14788
TSLP	Thymic stromal lymphopoietin	Q969D9
TWEAK	TNF-related weak inducer of apoptosis	O43508
uPA	Urokinase-type plasminogen activator	P00749
VEGFA	Vascular endothelial growth factor A	P15692

21

Table S3. Inflammation-related protein levels at 24 years according to body mass index trajectories.

Proteins*	Increasing - persistent high (n=35)	High - accelerated increasing (n=119)	Increasing - accelerated resolving (n=60)	Normal - above normal (n=419)	Stable normal (n=958)	Decreasing - persistent low (n=275)	p
CDCP1	1.02 ± 1.15	0.76 ± 1.07	-0.07 ± 0.98	-0.06 ± 0.97	-0.04 ± 0.96	-0.23 ± 0.94	< 0.001
MCP3	0.98 ± 0.74	0.70 ± 0.84	0.10 ± 0.85	-0.06 ± 0.99	-0.06 ± 0.98	-0.19 ± 0.97	< 0.001
IL6	0.90 ± 0.91	0.74 ± 0.82	0.11 ± 1.10	-0.10 ± 0.99	-0.05 ± 0.99	-0.14 ± 0.99	< 0.001
IL18R1	1.03 ± 0.92	0.70 ± 1.12	-0.12 ± 1.20	-0.09 ± 0.98	-0.04 ± 0.95	-0.10 ± 0.96	< 0.001
HGF	1.02 ± 0.95	0.60 ± 0.88	0.05 ± 0.86	-0.04 ± 1.04	-0.08 ± 0.95	-0.08 ± 1.01	< 0.001
VEGFA	0.80 ± 0.66	0.59 ± 0.95	-0.20 ± 0.97	-0.07 ± 1.03	-0.03 ± 0.97	-0.11 ± 0.98	< 0.001
CCL3	0.68 ± 0.76	0.62 ± 0.96	0.05 ± 1.11	-0.03 ± 1.01	-0.05 ± 0.99	-0.16 ± 0.94	< 0.001
FGF21	0.76 ± 0.88	0.47 ± 1.05	0.05 ± 1.08	-0.18 ± 0.99	0.01 ± 0.99	-0.02 ± 0.92	< 0.001
TRAIL	0.54 ± 0.82	0.39 ± 0.92	0.48 ± 0.98	0.05 ± 1.00	-0.06 ± 0.99	-0.20 ± 1.02	< 0.001
CCL4	0.50 ± 0.88	0.55 ± 0.81	-0.27 ± 1.03	-0.02 ± 1.01	-0.05 ± 0.98	-0.02 ± 0.98	< 0.001
IL18	0.65 ± 0.80	0.47 ± 1.01	-0.03 ± 0.97	-0.04 ± 1.01	0.01 ± 0.97	-0.17 ± 1.03	< 0.001
TNFSF14	0.78 ± 0.89	0.43 ± 0.87	-0.06 ± 0.92	-0.03 ± 1.05	-0.05 ± 0.95	-0.06 ± 0.97	< 0.001
FGF23	0.42 ± 0.69	0.53 ± 0.90	0.03 ± 1.19	-0.11 ± 0.97	-0.01 ± 1.00	-0.08 ± 1.02	< 0.001
MCP4	0.78 ± 0.78	0.36 ± 0.98	0.14 ± 0.97	-0.06 ± 1.03	-0.08 ± 0.97	0.01 ± 0.98	< 0.001
CCL19	0.61 ± 0.82	0.43 ± 0.90	0.00 ± 1.02	-0.13 ± 1.01	-0.00 ± 1.01	0.02 ± 0.96	< 0.001
MCP1	0.68 ± 0.83	0.39 ± 0.86	-0.04 ± 0.96	-0.04 ± 1.00	-0.07 ± 0.99	-0.01 ± 1.05	< 0.001
LAPTGFbeta1	0.76 ± 0.81	0.34 ± 0.94	-0.08 ± 1.09	-0.08 ± 1.02	-0.02 ± 0.97	-0.06 ± 0.98	< 0.001
IL10RB	0.64 ± 1.08	0.35 ± 0.93	-0.10 ± 1.05	-0.12 ± 1.00	-0.02 ± 0.99	0.03 ± 0.98	< 0.001
TRANCE	0.45 ± 0.87	0.34 ± 0.92	0.35 ± 0.94	0.02 ± 0.98	-0.05 ± 1.00	-0.10 ± 1.03	< 0.001
AXIN1	0.77 ± 0.89	0.29 ± 0.98	0.00 ± 1.09	-0.06 ± 1.04	-0.02 ± 0.97	-0.07 ± 1.03	< 0.001
OSM	0.66 ± 1.00	0.30 ± 0.88	-0.20 ± 0.95	0.04 ± 1.02	-0.06 ± 0.99	-0.00 ± 0.97	< 0.001
SIRT2	0.74 ± 0.95	0.28 ± 0.99	-0.07 ± 1.02	-0.06 ± 1.04	-0.01 ± 0.98	-0.05 ± 1.00	< 0.001
STAMBP	0.74 ± 0.92	0.27 ± 1.00	-0.04 ± 1.03	-0.05 ± 1.02	-0.02 ± 0.97	-0.03 ± 1.00	< 0.001
CD40	0.72 ± 0.85	0.26 ± 1.04	0.05 ± 1.03	-0.05 ± 1.02	-0.02 ± 0.96	-0.07 ± 1.02	< 0.001
Flt3L	0.18 ± 0.90	0.18 ± 1.08	-0.02 ± 0.88	-0.21 ± 0.95	0.02 ± 0.98	0.11 ± 1.07	< 0.001
ADA	0.55 ± 0.77	0.30 ± 0.91	0.10 ± 1.02	0.01 ± 1.02	-0.01 ± 0.99	-0.14 ± 0.96	< 0.001

IL22RA1	0.61 ± 0.78	0.31 ± 0.91	-0.08 ± 0.97	-0.03 ± 1.00	-0.05 ± 1.01	-0.01 ± 0.95	< 0.001
4EBP1	0.70 ± 0.95	0.27 ± 0.97	-0.06 ± 0.96	-0.03 ± 1.09	-0.03 ± 0.97	-0.02 ± 0.99	< 0.001
IL7	0.63 ± 0.81	0.28 ± 0.93	-0.13 ± 1.04	-0.02 ± 0.99	-0.03 ± 0.99	-0.08 ± 0.96	< 0.001
CSF1	0.30 ± 1.02	0.40 ± 0.99	-0.14 ± 0.97	-0.05 ± 1.03	0.01 ± 0.98	-0.07 ± 1.01	< 0.001
IL12B	0.28 ± 1.04	0.38 ± 1.03	-0.08 ± 0.99	-0.08 ± 1.06	0.00 ± 0.98	-0.01 ± 0.93	< 0.001
IL8	0.49 ± 0.78	0.31 ± 1.01	-0.08 ± 1.01	-0.07 ± 1.02	-0.01 ± 0.99	-0.03 ± 0.98	< 0.001
CXCL5	0.52 ± 0.92	0.27 ± 0.95	0.10 ± 1.04	-0.03 ± 1.07	-0.03 ± 0.98	-0.05 ± 0.98	< 0.001
ENRAGE	0.43 ± 0.90	0.14 ± 0.89	0.05 ± 0.89	0.12 ± 1.05	-0.05 ± 1.01	-0.11 ± 0.95	0.001
CCL20	0.38 ± 0.97	0.26 ± 1.00	0.11 ± 1.04	-0.12 ± 1.03	0.02 ± 0.98	0.02 ± 1.00	0.001
CXCL6	0.53 ± 1.05	0.24 ± 0.98	-0.02 ± 0.93	0.00 ± 1.01	-0.03 ± 0.99	-0.06 ± 0.98	0.002
CD5	0.28 ± 0.87	0.33 ± 0.96	-0.01 ± 0.88	-0.03 ± 1.09	-0.04 ± 0.95	-0.06 ± 1.03	0.002
SCF	-0.20 ± 0.95	-0.33 ± 1.04	0.10 ± 0.77	0.07 ± 1.01	-0.02 ± 0.99	0.04 ± 1.05	0.003
IL10	0.33 ± 0.89	0.18 ± 0.96	-0.05 ± 1.15	-0.04 ± 1.03	0.03 ± 0.99	-0.17 ± 0.96	0.005
IL2	0.27 ± 1.01	0.25 ± 1.06	-0.20 ± 1.12	-0.02 ± 0.96	0.00 ± 0.99	-0.10 ± 0.95	0.009
CASP8	0.46 ± 0.92	0.12 ± 0.94	-0.16 ± 0.91	0.06 ± 1.08	-0.03 ± 0.96	-0.09 ± 1.02	0.009
ST1A1	0.58 ± 1.06	0.10 ± 1.00	-0.09 ± 1.04	-0.03 ± 1.02	0.00 ± 0.99	-0.06 ± 1.02	0.013
CXCL10	0.32 ± 0.81	0.24 ± 0.96	0.08 ± 0.93	0.00 ± 1.00	0.00 ± 1.03	-0.12 ± 0.96	0.014
IL20	0.17 ± 0.84	0.27 ± 0.97	-0.07 ± 1.00	0.06 ± 0.97	-0.05 ± 1.03	-0.03 ± 0.99	0.016
MCP2	0.45 ± 0.90	0.15 ± 1.03	0.12 ± 0.96	0.00 ± 1.01	-0.02 ± 0.98	-0.10 ± 1.03	0.019
IL5	0.14 ± 1.06	0.22 ± 0.97	0.18 ± 0.96	0.04 ± 1.01	-0.02 ± 1.01	-0.12 ± 0.94	0.019
CXCL1	0.57 ± 0.84	0.12 ± 1.00	0.03 ± 0.96	-0.02 ± 1.04	-0.01 ± 1.00	-0.04 ± 1.00	0.02
SLAMF1	0.14 ± 1.06	0.19 ± 1.02	0.17 ± 1.03	0.01 ± 0.94	-0.00 ± 1.01	-0.15 ± 1.02	0.022
IL33	0.19 ± 0.96	0.26 ± 0.98	-0.03 ± 1.13	0.01 ± 1.00	-0.05 ± 0.98	0.03 ± 1.01	0.027
IL17A	0.06 ± 1.01	0.13 ± 0.98	-0.07 ± 0.96	-0.13 ± 0.95	0.06 ± 1.04	-0.01 ± 0.95	0.028
CCL28	-0.21 ± 0.86	-0.23 ± 1.02	0.04 ± 0.93	-0.06 ± 1.02	0.02 ± 1.00	0.10 ± 1.04	0.031
TNFRSF9	0.32 ± 0.91	0.22 ± 0.98	0.04 ± 1.06	-0.05 ± 1.03	0.01 ± 0.99	-0.06 ± 0.98	0.044
PDL1	0.29 ± 0.97	0.21 ± 1.02	0.11 ± 1.02	-0.05 ± 0.98	0.01 ± 0.97	-0.07 ± 1.07	0.047
LIF	-0.06 ± 0.98	0.27 ± 0.93	0.08 ± 1.06	-0.01 ± 1.03	-0.02 ± 0.98	-0.06 ± 1.00	0.055
CST5	-0.13 ± 0.94	-0.25 ± 0.99	0.14 ± 0.93	-0.02 ± 1.00	-0.00 ± 1.00	0.08 ± 1.02	0.06
TNF	0.48 ± 0.95	0.07 ± 0.89	0.10 ± 1.15	-0.02 ± 1.07	-0.01 ± 0.96	-0.06 ± 1.03	0.062

CD244	0.45 ± 0.77	0.08 ± 1.10	-0.02 ± 1.00	-0.04 ± 0.97	0.01 ± 0.97	-0.06 ± 1.08	0.074
FGF19	-0.18 ± 0.95	-0.22 ± 1.10	-0.08 ± 0.91	-0.06 ± 0.99	0.01 ± 1.00	0.08 ± 1.02	0.076
TWEAK	0.04 ± 0.87	-0.14 ± 0.93	-0.02 ± 0.91	0.08 ± 1.05	-0.06 ± 0.98	0.08 ± 1.07	0.077
CD6	0.33 ± 0.84	0.16 ± 0.96	-0.06 ± 0.92	0.01 ± 1.05	-0.04 ± 0.97	-0.03 ± 1.01	0.111
IL17C	0.23 ± 0.71	0.16 ± 0.95	0.14 ± 1.05	-0.00 ± 0.98	0.02 ± 1.02	-0.10 ± 0.99	0.113
FGF5	-0.01 ± 0.96	-0.15 ± 1.09	-0.01 ± 1.04	-0.04 ± 0.93	-0.02 ± 0.99	0.14 ± 1.05	0.114
DNER	0.08 ± 1.06	-0.21 ± 0.91	0.17 ± 0.91	-0.07 ± 1.05	-0.00 ± 0.99	0.02 ± 0.97	0.116
CXCL11	0.10 ± 0.78	0.13 ± 1.10	0.01 ± 1.04	-0.05 ± 1.00	0.05 ± 0.99	-0.11 ± 0.99	0.123
IL1alpha	0.15 ± 1.04	0.07 ± 1.01	0.13 ± 1.04	0.05 ± 1.01	0.00 ± 0.99	-0.13 ± 0.99	0.143
CCL23	-0.06 ± 0.83	-0.10 ± 1.05	-0.27 ± 0.98	-0.01 ± 0.98	0.05 ± 1.01	0.01 ± 0.99	0.157
uPA	0.05 ± 0.76	0.12 ± 0.96	0.24 ± 1.02	0.01 ± 1.01	-0.05 ± 1.00	-0.01 ± 1.04	0.183
ARTN	-0.14 ± 0.83	0.18 ± 1.09	-0.05 ± 0.85	-0.08 ± 1.00	-0.01 ± 1.00	-0.06 ± 0.99	0.2
CCL11	0.27 ± 0.81	-0.09 ± 0.92	0.16 ± 0.93	0.03 ± 1.01	-0.05 ± 1.01	0.00 ± 1.03	0.203
IL15RA	0.10 ± 1.01	0.17 ± 1.00	0.04 ± 1.00	-0.01 ± 0.95	-0.01 ± 1.00	-0.10 ± 1.07	0.232
CX3CL1	-0.06 ± 0.88	-0.18 ± 1.00	0.20 ± 1.13	0.00 ± 1.06	-0.01 ± 0.96	0.04 ± 1.07	0.246
IL10RA	0.03 ± 0.87	0.07 ± 0.80	-0.14 ± 1.03	0.07 ± 1.02	0.01 ± 0.96	-0.10 ± 1.13	0.248
LIFR	-0.07 ± 0.87	-0.17 ± 0.99	0.12 ± 1.05	0.04 ± 0.99	-0.01 ± 1.00	-0.08 ± 1.08	0.268
IL20RA	0.12 ± 0.95	0.13 ± 0.97	0.11 ± 0.95	0.05 ± 0.98	-0.05 ± 1.00	0.02 ± 1.09	0.274
IFNgamma	0.04 ± 0.80	0.09 ± 0.91	0.09 ± 1.11	-0.01 ± 0.99	0.04 ± 1.04	-0.11 ± 0.94	0.294
IL2RB	-0.25 ± 1.10	0.01 ± 0.86	-0.10 ± 1.01	0.07 ± 1.01	0.01 ± 0.99	-0.06 ± 1.07	0.35
IL13	0.13 ± 0.80	0.08 ± 0.93	-0.08 ± 0.99	0.02 ± 0.94	-0.00 ± 1.01	-0.11 ± 1.08	0.382
TGFalpha	0.08 ± 1.02	0.11 ± 0.95	-0.23 ± 0.90	-0.02 ± 1.06	-0.01 ± 0.96	0.03 ± 1.01	0.394
IL24	0.04 ± 1.06	0.17 ± 1.01	-0.06 ± 0.96	-0.06 ± 1.03	0.01 ± 1.00	0.01 ± 0.99	0.409
OPG	0.02 ± 0.98	0.12 ± 0.96	-0.09 ± 0.92	-0.06 ± 0.94	0.01 ± 1.05	0.06 ± 1.01	0.429
MMP1	0.25 ± 0.88	0.12 ± 1.08	-0.05 ± 1.01	-0.02 ± 1.01	-0.02 ± 0.97	-0.02 ± 1.02	0.432
IL4	-0.00 ± 1.09	0.07 ± 1.06	-0.19 ± 0.96	-0.02 ± 0.97	0.03 ± 0.99	-0.00 ± 1.08	0.572
MMP10	0.07 ± 0.99	-0.08 ± 0.94	0.17 ± 1.21	-0.03 ± 1.02	0.01 ± 0.98	-0.05 ± 0.96	0.632
NRTN	0.19 ± 0.90	0.05 ± 0.85	-0.17 ± 1.10	0.01 ± 0.99	-0.01 ± 1.02	0.00 ± 1.04	0.65
TNFB	-0.08 ± 1.04	-0.01 ± 0.94	-0.04 ± 0.94	-0.06 ± 1.03	0.02 ± 1.00	0.04 ± 1.06	0.702
TSLP	-0.02 ± 1.16	0.12 ± 0.97	0.10 ± 1.07	-0.04 ± 0.99	0.01 ± 1.02	-0.02 ± 0.98	0.707

GDNF	-0.03 ± 0.71	-0.06 ± 0.87	0.19 ± 0.88	-0.03 ± 0.99	-0.01 ± 1.02	-0.01 ± 1.10	0.717
NT3	0.01 ± 1.07	-0.01 ± 1.03	0.07 ± 0.99	-0.07 ± 1.01	-0.02 ± 0.99	0.04 ± 1.06	0.768
CD8A	0.06 ± 0.99	-0.04 ± 0.96	0.13 ± 1.13	0.00 ± 0.94	-0.00 ± 0.98	-0.07 ± 1.12	0.776
BetaNGF	-0.05 ± 0.87	0.02 ± 1.01	0.09 ± 1.02	0.04 ± 0.98	-0.01 ± 0.99	-0.05 ± 1.10	0.844
CXCL9	0.02 ± 0.85	0.03 ± 0.98	0.04 ± 1.12	0.00 ± 0.97	0.03 ± 1.02	-0.07 ± 0.93	0.845
CCL25	-0.15 ± 0.77	-0.02 ± 1.14	0.05 ± 1.14	0.02 ± 0.96	-0.01 ± 1.00	0.01 ± 1.03	0.932

*: Protein concentrations were normalized based on inverse normal transformation.\cite{RN902}

Table S4. Characteristics of the included and excluded participants.

	Excluded participants (n=885)	Included participants (n=3204)	p
Sex			0.321
Male	460 (52.0%)	1605 (50.1%)	
Female	425 (48.0%)	1599 (49.9%)	
Preterm birth	45 (5.1%)	182 (5.7%)	0.493
Birth weight			
Kilogram	3.52 ± 0.54	3.53 ± 0.56	0.665
Z scores	0.42 ± 1.14	0.45 ± 1.18	0.575
Cesarean section	98 (11.1%)	409 (12.8%)	0.176
Exclusive breast feeding >= 4 months	569 (73.4%)	2547 (81.0%)	<0.001
Parental education			<0.001
Primary school/high school	464 (52.7%)	1457 (45.5%)	
University	417 (47.3%)	1744 (54.5%)	
Maternal characters			
Smoking during pregnancy	138 (15.6%)	389 (12.1%)	0.006
Age at delivery	29.71 ± 4.50	30.49 ± 4.50	<0.001
BMI at early pregnancy	23.03 ± 3.62	22.91 ± 3.25	0.361
Diabetes mellitus	14 (1.6%)	40 (1.3%)	0.438
Hypertension	25 (2.9%)	93 (3.0%)	0.908
Parity before the index person was born			0.007
One	296 (34.1%)	960 (30.5%)	
Two or more	97 (11.2%)	285 (9.1%)	

BMI=body mass index. The results were illustrated with mean ± standard deviation, or numbers (proportions), respectively.

Table S5. The absolute error loss across latent class mixture models incorporating linear, quadratic, and cubic terms.

	Models incorporated with linear terms		Models incorporated with quadratic terms		Models incorporated with cubic terms	
Number of trajectories	Mean	SD	Mean	SD	Mean	SD
2	0.760	0.010	0.715	0.006	0.720	0.006
3	0.736	0.014	0.690	0.007	0.692	0.005
4	0.716	0.014	0.678	0.008	0.686	0.005
5	0.715	0.008	0.661	0.004	0.674	0.004
6	0.704	0.014	0.653	0.005	0.656	0.006
7	0.675	0.051	0.647	0.006	0.647	0.007

SD: standard deviation.

Table S6. The results of Latent Class Mixture models incorporating quadratic terms.

Number of latent classes	log-likelihood	BIC	Percentage (%) of participants per class	Mean posterior probabilities
1	-39763.89	79632.71	100	NA
2	-38560.34	77265.97	33.83/66.17	0.89/0.92
3	-38349.87	76885.40	36.33/11.05/52.62	0.83/0.85/0.80
4	-38269.34	76764.69	34.46/51.09/3.78/10.67	0.84/0.80/0.76/0.83
5	-38206.89	76680.17	4.9/52.31/21.47/16.04/5.27	0.82/0.78/0.74/0.78/0.74
6	-38160.80	76628.34	4.43/6.52/14.04/2.31/22.5/50.19	0.76/0.72/0.80/0.79/0.73/0.78
7	-38142.43	76631.97	49.63/7.46/0.75/3.96/21.97/14.11/2.12	0.79/0.73/0.84/0.74/0.73/0.80/0.73

BIC=Bayesian information criterion.

Table S7. The mean and standard deviation of BMI and BMI z-scores for all the follow-up points for the BMI groups.

Age, Year	0	0.5	1	1.5	2	3	4	5	7	8	10	12	16	24
BMI, z scores														
Increasing - persistent high	-0.07 ± 1.43	-0.38 ± 1.00	0.10 ± 1.01	0.36 ± 0.83	0.63 ± 0.95	0.81 ± 1.11	1.33 ± 1.29	1.42 ± 1.11	1.95 ± 1.17	2.20 ± 0.85	2.18 ± 0.62	1.99 ± 0.60	2.22 ± 0.69	2.65 ± 1.41
High - accelerated increasing	0.89 ± 0.89	0.39 ± 1.03	0.76 ± 0.98	0.86 ± 1.03	1.11 ± 1.04	0.97 ± 1.13	1.09 ± 0.90	0.83 ± 0.90	0.96 ± 0.85	1.33 ± 0.73	1.36 ± 0.68	1.01 ± 0.69	1.45 ± 0.59	1.93 ± 0.94
Increasing - accelerated resolving	-0.71 ± 1.70	-0.46 ± 0.94	0.06 ± 0.92	0.34 ± 0.87	0.44 ± 0.98	1.12 ± 1.24	1.27 ± 1.11	1.56 ± 1.21	1.92 ± 0.95	2.04 ± 0.75	1.97 ± 0.63	1.49 ± 0.69	1.22 ± 0.73	0.36 ± 0.63
Normal - above normal	0.28 ± 1.10	-0.04 ± 0.97	0.39 ± 0.90	0.58 ± 0.90	0.79 ± 0.91	0.73 ± 0.95	1.02 ± 0.85	0.87 ± 0.84	1.08 ± 0.80	1.26 ± 0.67	1.14 ± 0.62	0.71 ± 0.64	0.56 ± 0.59	0.08 ± 0.60
Stable normal	0.43 ± 1.12	-0.05 ± 1.00	0.31 ± 0.93	0.43 ± 0.95	0.53 ± 0.94	0.35 ± 0.87	0.45 ± 0.73	0.09 ± 0.75	0.07 ± 0.70	0.27 ± 0.63	0.01 ± 0.65	-0.38 ± 0.68	-0.14 ± 0.67	-0.26 ± 0.60
Decreasing - persistent low	0.82 ± 0.94	0.08 ± 1.04	0.36 ± 0.96	0.40 ± 0.92	0.38 ± 0.86	0.00 ± 0.93	-0.01 ± 0.69	-0.46 ± 0.75	-0.72 ± 0.73	-0.53 ± 0.61	-0.92 ± 0.64	-1.42 ± 0.76	-1.00 ± 0.71	-0.71 ± 0.66
BMI, kg/m2														
Increasing - persistent high	13.42 ± 1.75	16.71 ± 1.35	16.86 ± 1.36	16.52 ± 1.11	16.54 ± 1.31	16.71 ± 1.59	17.43 ± 2.18	17.64 ± 2.09	19.65 ± 3.11	21.31 ± 2.98	22.99 ± 3.15	25.28 ± 3.05	30.31 ± 4.29	33.66 ± 5.43
High - accelerated increasing	14.60 ± 1.21	17.78 ± 1.65	17.76 ± 1.57	17.22 ± 1.56	17.24 ± 1.67	16.91 ± 1.72	16.96 ± 1.48	16.61 ± 1.57	17.33 ± 1.87	18.89 ± 2.12	20.12 ± 2.22	21.77 ± 2.71	26.39 ± 2.89	30.80 ± 3.62
Increasing - accelerated resolving	12.67 ± 1.90	16.58 ± 1.36	16.79 ± 1.27	16.50 ± 1.19	16.27 ± 1.34	17.15 ± 1.83	17.22 ± 1.83	17.81 ± 2.23	19.30 ± 2.34	20.57 ± 2.33	22.00 ± 2.37	23.07 ± 2.72	25.31 ± 2.93	24.80 ± 2.52
Normal - above normal	13.82 ± 1.39	17.21 ± 1.78	17.23 ± 1.34	16.82 ± 1.32	16.73 ± 1.32	16.56 ± 1.35	16.82 ± 1.36	16.62 ± 1.40	17.50 ± 1.70	18.53 ± 1.83	19.28 ± 1.75	20.39 ± 1.94	22.80 ± 1.98	23.64 ± 2.36

Stable normal	14.01 ± 1.52	17.14 ± 1.57	17.08 ± 1.36	16.60 ± 1.36	16.35 ± 1.29	16.02 ± 1.18	15.94 ± 1.05	15.43 ± 1.09	15.65 ± 1.14	16.40 ± 1.22	16.67 ± 1.26	17.73 ± 1.43	20.75 ± 1.90	22.27 ± 2.36
Decreasing - persistent low	14.52 ± 1.28	17.30 ± 1.62	17.15 ± 1.44	16.52 ± 1.32	16.12 ± 1.19	15.59 ± 1.29	15.30 ± 0.98	14.65 ± 0.98	14.48 ± 0.92	15.07 ± 0.90	15.06 ± 0.93	15.86 ± 1.15	18.61 ± 1.55	20.50 ± 2.60

The results were illustrated with mean ± standard deviation.

Table S8. Characteristics of males in each body mass index trajectory.

Characteristics	Increasing - persistent high (n=45)	High - accelerated increasing (n=95)	Increasing - accelerated resolving (n=92)	Normal - above normal (n=407)	Stable normal (n=764)	Decreasing - persistent low (n=202)	p
Preterm birth	6 (13.3%)*	3 (3.2%)	13 (14.1%)***	27 (6.6%)	38 (5.0%)	5 (2.5%)	< 0.001#
Birth weight							
Kilogram	3.32 ± 0.61**	3.77 ± 0.42**	3.28 ± 0.66***	3.56 ± 0.57	3.59 ± 0.56)	3.75 ± 0.53***	< 0.001
Z scores	-0.16 ± 1.29**	0.81 ± 0.78**	-0.23 ± 1.49***	0.38 ± 1.18	0.44 ± 1.12	0.75 ± 1.00***	< 0.001
Cesarean section	10 (22.2%)*	12 (12.6%)	20 (21.7%)**	63 (15.5%)*	87 (11.4%)	28 (13.9%)	0.029
Exclusive breastfeeding >= 4 months	39 (86.7%)	75 (80.6%)	68 (76.4%)	320 (79.8%)	618 (82.6%)	168 (84.4%)	0.432
Current smoking, Yes							
24 years of age	6 (14.0%)	20 (23.5%)	12 (20.0%)	58 (18.1%)	113 (19.1%)	18 (11.1%)	0.119
26 years of age	0 (0.0%)	3 (23.1%)	1 (5.6%)	9 (9.0%)	19 (10.7%)	2 (4.4%)	0.399#
Parental education		**					0.023
Primary school/high school	20 (44.4%)	56 (58.9%)	46 (50.0%)	193 (47.4%)	320 (41.9%)	81 (40.1%)	
University	25 (55.6%)	39 (41.1%)	46 (50.0%)	214 (52.6%)	444 (58.1%)	121 (59.9%)	
Maternal characteristics during pregnancy							
Smoking during pregnancy	5 (11.1%)	14 (14.7%)	19 (20.7%)**	54 (13.3%)	84 (11.0%)	22 (10.9%)	0.134
Age at delivery	29.80 ± 5.55	29.56 ± 4.24	30.46 ± 4.45	30.67 ± 4.72	30.43 ± 4.42	30.61 ± 4.35	0.335
BMI at early pregnancy	25.81 ± 4.57***	25.49 ± 4.49***	24.63 ± 4.08***	23.43 ± 3.19***	22.36 ± 2.72	21.82 ± 2.92*	< 0.001
Diabetes mellitus	1 (2.2%)	0 (0.0%)	3 (3.4%)	8 (2.0%)	7 (0.9%)	4 (2.0%)	0.159#
Hypertension	2 (4.4%)	2 (2.1%)	7 (7.9%)**	15 (3.7%)	16 (2.1%)	4 (2.0%)	0.057#
Parity before the index person was born							0.485#
One	13 (28.9%)	35 (37.2%)	25 (28.1%)	116 (28.8%)	243 (32.4%)	66 (33.3%)	
Two or more	2 (4.4%)	11 (11.7%)	4 (4.5%)	34 (8.4%)	65 (8.7%)	14 (7.1%)	

BMI=body mass index.

The results were illustrated with mean ± standard deviation, or numbers (proportions), respectively.

* p<0.05; ** p<0.01; *** p<0.001 for comparisons between each trajectory group and the stable normal group.

# Based on Fisher's exact test.

Table S9. Characteristics of females according to body mass index trajectories.

Characteristics	Increasing - persistent high (n=29)	High - accelerated increasing (n=114)	Increasing - accelerated resolving (n=50)	Normal - above normal (n=314)	Stable normal (n=844)	Decreasing - persistent low (n=248)	p
Preterm birth	4 (13.8%)	4 (3.5%)	13 (26.0%)***	21 (6.7%)	42 (5.0%)	6 (2.4%)	< 0.001#
Birth weight							
Kilogram	3.36 ± 0.68	3.63 ± 0.49**	2.75 ± 0.82***	3.42 ± 0.54	3.47 ± 0.52	3.63 ± 0.49***	< 0.001
Z scores	0.17 ± 1.56	0.79 ± 1.01**	-1.22 ± 1.96***	0.36 ± 1.16	0.47 ± 1.14	0.81 ± 1.01***	< 0.001
Cesarean section	5 (17.2%)	17 (14.9%)	11 (22.0%)*	45 (14.3%)	87 (10.3%)	24 (9.7%)	0.039
Exclusive breast feeding >= 4 months	20 (74.1%)	86 (77.5%)	35 (71.4%)	241 (79.3%)	670 (80.4%)	207 (84.5%)	0.255
Current smoking, Yes							
24 years of age	5 (17.9%)	27 (25.2%)	8 (21.1%)	58 (20.9%)	163 (21.6%)	45 (21.4%)	0.948
26 years of age	1 (14.3%)	5 (12.2%)	1 (11.1%)	12 (11.1%)	49 (15.5%)	10 (9.4%)	0.618#
Parental education		*					0.233
Primary school/high school	15 (51.7%)	65 (57.0%)	19 (38.0%)	134 (42.8%)	387 (46.0%)	121 (48.8%)	
University	14 (48.3%)	49 (43.0%)	31 (62.0%)	179 (57.2%)	455 (54.0%)	127 (51.2%)	
Maternal characters during pregnancy							
Smoking during pregnancy	5 (17.9%)	26 (22.8%)***	3 (6.0%)	46 (14.6%)*	88 (10.4%)	23 (9.3%)	0.003#
Age at delivery	32.56 ± 5.58*	29.86 ± 5.02	32.18 ± 4.96**	30.67 ± 4.57	30.48 ± 4.37	30.35 ± 4.24	0.009
BMI at early pregnancy	27.60 ± 4.64***	24.77 ± 4.05***	23.71 ± 3.39***	23.40 ± 2.96***	22.46 ± 2.87	21.70 ± 2.58***	< 0.001
Diabetes mellitus	1 (3.7%)	0 (0.0%)	1 (2.0%)	5 (1.6%)	7 (0.8%)	3 (1.2%)	0.259#
Hypertension	3 (11.1%)	4 (3.6%)	3 (6.1%)	10 (3.3%)	26 (3.1%)	1 (0.4%)*	0.011#
Parity before the index person was born	***					**	< 0.001#
One	4 (14.8%)	29 (25.9%)	15 (30.6%)	79 (25.9%)	236 (28.5%)	99 (40.6%)	
Two or more	9 (33.3%)	14 (12.5%)	4 (8.2%)	38 (12.5%)	69 (8.3%)	21 (8.6%)	

BMI=body mass index.
The results were illustrated with mean ± standard deviation, or numbers (proportions), respectively.
* p<0.05; ** p<0.01; *** p<0.001 for comparisons between each trajectory group and the stable normal group.
# Based on Fisher's exact test.

Table S10. Bioimpedance at 24 years and 26 years according to body mass index trajectories. *

	Increasing - persistent high	High - accelerated increasing	Increasing - accelerated resolving	Normal - above normal	Stable normal	Decreasing - persistent low	p
All at 24 years of age*	n=40	n=131	n=62	n=437	n=986	n=290	
Fat mass index, kg/m2	11.40 ± 3.10	11.51 ± 2.89	8.12 ± 2.67	7.39 ± 2.69	7.11 ± 2.74	6.37 ± 2.76	< 0.001
Fat-free mass index, kg/m2	22.14 ± 2.20	20.42 ± 2.19	18.80 ± 2.11	18.29 ± 1.93	17.40 ± 1.84	16.42 ± 1.79	< 0.001
Male at 24 years of age*	n=25	n=50	n=33	n=227	n=419	n=120	
Fat mass index, kg/m2	9.56 ± 2.16	8.61 ± 1.96	6.22 ± 1.89	5.36 ± 1.52	4.59 ± 1.55	3.88 ± 1.47	< 0.001
Fat-free mass index, kg/m2	23.17 ± 1.68	22.32 ± 1.64	20.41 ± 1.24	19.70 ± 1.35	18.96 ± 1.37	17.91 ± 1.43	< 0.001
Female at 24 years of age*	n=15	n=81	n=29	n=210	n=567	n=170	
Fat mass index, kg/m2	14.47 ± 1.62	13.31 ± 1.65	10.29 ± 1.53	9.59 ± 1.81	8.97 ± 1.78	8.12 ± 2.01	< 0.001
Fat-free mass index, kg/m2	20.43 ± 1.89	19.25 ± 1.57	16.96 ± 1.19	16.77 ± 1.13	16.24 ± 1.17	15.37 ± 1.15	< 0.001
All at 26 years of age#	n=15	n=51	n=27	n=205	n=484	n=149	
Fat mass index, kg/m2	11.92 ± 5.74	11.30 ± 4.50	6.19 ± 2.60	5.83 ± 2.20	5.12 ± 1.94	4.51 ± 1.67	< 0.001
Fat-free mass index, kg/m2	22.25 ± 2.62	20.27 ± 2.31	19.74 ± 2.13	18.43 ± 1.99	17.36 ± 1.85	16.28 ± 1.70	< 0.001
Male at 26 years of age#	n=9	n=13	n=18	n=101	n=175	n=45	
Fat mass index, kg/m2	10.15 ± 5.47	7.89 ± 2.61	5.77 ± 2.94	4.86 ± 1.78	3.99 ± 1.61	3.53 ± 1.50	< 0.001
Fat-free mass index, kg/m2	22.49 ± 2.26	21.73 ± 1.40	20.98 ± 1.25	19.92 ± 1.47	19.07 ± 1.41	18.15 ± 1.38	< 0.001
Female at 26 years of age#	n=6	n=38	n=9	n=104	n=309	n=104	
Fat mass index, kg/m2	14.58 ± 5.49	12.47 ± 4.44	7.03 ± 1.55	6.78 ± 2.16	5.76 ± 1.82	4.94 ± 1.56	< 0.001
Fat-free mass index, kg/m2	21.90 ± 3.28	19.78 ± 2.36	17.26 ± 1.00	16.98 ± 1.20	16.40 ± 1.28	15.47 ± 1.08	< 0.001

*: Only participants with blood lipid test at 24 years of age were included.

#: Only participants with HbA1c test at 26 years of age were included.

Table S11. Mean differences and 95% confidence intervals for the associations of body mass index trajectories with cardiometabolic profile at late adolescence and young adulthood by linear regression. The stable normal group was the reference group.

	Increasing - persistent high		High - accelerated increasing		Increasing - accelerated resolving		Normal - above normal		Decreasing - persistent low	
Models	1	2	1	2	1	2	1	2	1	2
BP at 24 years										
Systolic BP, mmHg	3.01 [-0.32, 6.37]	2.08 [-1.56, 5.82]	**3.44 [1.41, 5.51]**	**2.51 [0.37, 4.84]**	1.54 [-1.16, 4.40]	1.26 [-1.71, 4.48]	0.23 [-0.95, 1.45]	0.07 [-1.21, 1.40]	**-1.67 [-2.82, -0.27]**	**-1.66 [-2.92, -0.16]**
Diastolic BP, mmHg	**4.67 [2.29, 7.03]**	**4.42 [1.80, 6.98]**	**2.16 [0.67, 3.62]**	1.39 [-0.39, 2.93]	0.78 [-1.43, 2.83]	0.66 [-1.70, 2.94]	-0.39 [-1.33, 0.66]	-0.39 [-1.40, 0.75]	-0.38 [-1.44, 0.84]	-0.28 [-1.43, 1.04]
Blood lipid at 24 years										
Triglyceride, mmol/L	**0.49 [0.27, 0.75]**	**0.48 [0.25, 0.76]**	**0.35 [0.24, 0.48]**	**0.32 [0.21, 0.45]**	**-0.14 [-0.21, -0.04]**	**-0.14 [-0.22, -0.03]**	-0.02 [-0.07, 0.03]	-0.01 [-0.07, 0.04]	0.00 [-0.05, 0.05]	0.02 [-0.05, 0.06]
Total cholesterol, mmol/L	**0.42 [0.18, 0.69]**	**0.30 [0.04, 0.56]**	**0.23 [0.09, 0.36]**	**0.16 [0.02, 0.31]**	0.01 [-0.17, 0.20]	-0.01 [-0.22, 0.19]	0.05 [-0.03, 0.13]	0.05 [-0.04, 0.14]	0.02 [-0.07, 0.11]	0.01 [-0.10, 0.11]
HDL, mmol/L	**-0.30 [-0.38, -0.21]**	**-0.30 [-0.39, -0.21]**	**-0.32 [-0.37, -0.27]**	**-0.31 [-0.37, -0.26]**	-0.02 [-0.12, 0.07]	-0.02 [-0.13, 0.08]	0.01 [-0.03, 0.05]	0.01 [-0.04, 0.05]	-0.01 [-0.06, 0.04]	-0.01 [-0.07, 0.04]
LDL, mmol/L	**0.41 [0.19, 0.65]**	**0.29 [0.05, 0.53]**	**0.36 [0.24, 0.49]**	**0.31 [0.18, 0.45]**	0.11 [-0.07, 0.28]	0.09 [-0.10, 0.28]	0.05 [-0.02, 0.13]	0.06 [-0.02, 0.15]	0.03 [-0.06, 0.12]	0.01 [-0.08, 0.11]
LDL/HDL ratio	**0.58 [0.34, 0.84]**	**0.48 [0.23, 0.74]**	**0.65 [0.52, 0.79]**	**0.61 [0.46, 0.75]**	0.09 [-0.06, 0.26]	0.07 [-0.09, 0.26]	0.03 [-0.03, 0.10]	0.04 [-0.03, 0.12]	0.03 [-0.05, 0.11]	0.02 [-0.06, 0.11]
Triglyceride/HDL ratio	**0.51 [0.31, 0.77]**	**0.51 [0.30, 0.79]**	**0.46 [0.34, 0.58]**	**0.42 [0.31, 0.56]**	-0.08 [-0.15, 0.00]	-0.08 [-0.15, 0.01]	-0.02 [-0.05, 0.02]	-0.01 [-0.05, 0.03]	0.00 [-0.04, 0.05]	0.01 [-0.03, 0.06]
Leukocyte at 24 years, 10^9/L	**1.11 [0.53, 1.75]**	**0.98 [0.37, 1.66]**	**0.85 [0.53, 1.19]**	**0.79 [0.45, 1.15]**	-0.33 [-0.69, 0.05]	-0.28 [-0.68, 0.14]	**0.23 [0.05, 0.41]**	**0.27 [0.07, 0.47]**	-0.01 [-0.21, 0.19]	-0.04 [-0.25, 0.18]
HbA1c at 26 years										
HbA1c, mmol/mol	**3.10 [1.86, 4.41]**	**3.61 [2.17, 5.54]**	**0.94 [0.25, 1.64]**	**1.18 [0.40, 1.98]**	0.04 [-0.89, 0.97]	-0.22 [-1.29, 0.85]	0.04 [-0.35, 0.43]	0.13 [-0.29, 0.56]	0.16 [-0.27, 0.60]	0.04 [-0.42, 0.51]

BP=blood pressure; HDL=high-density lipoprotein; LDL=low-density lipoprotein. HbA1c= Hemoglobin A1C.
Model 1 was adjusted for age and sex.
Model 2 was additionally adjusted for smoking status, parental education, maternal smoking during pregnancy, maternal body mass index at early pregnancy, maternal hypertension, parity before the index person was born, and caesarean section based on model 1.

Table S12. Mean differences and 95% confidence intervals for the associations of body mass index trajectories with cardiometabolic profile at late adolescence and young adulthood by linear regression in males. The stable normal group was the reference group.

	Increasing - persistent high		High - accelerated increasing		Increasing - accelerated resolving		Normal - above normal		Decreasing - persistent low	
Models	1	2	1	2	1	2	1	2	1	2
BP at 24 years										
Systolic BP, mmHg	1.97 [-2.25, 6.32]	1.18 [-3.15, 6.02]	**3.25 [0.06, 6.35]**	3.22 [-0.21, 6.63]	0.93 [-2.54, 4.87]	0.2 [-3.45, 4.42]	1.12 [-0.54, 2.92]	1.09 [-0.74, 3.06]	-1.89 [-3.66, 0.25]	-1.69 [-3.69, 0.67]
Diastolic BP, mmHg	**4.54 [1.40, 7.61]**	**3.79 [0.14, 7.05]**	**3.59 [1.27, 5.81]**	2.27 [-0.43, 4.68]	0.70 [-2.22, 3.52]	-0.49 [-3.60, 2.71]	-0.35 [-1.65, 1.17]	-0.39 [-1.77, 1.23]	0.41 [-1.36, 2.13]	0.76 [-1.22, 2.58]
Blood lipid at 24 years										
Triglyceride, mmol/L	**0.59 [0.29, 0.98]**	**0.57 [0.25, 0.99]**	**0.41 [0.23, 0.64]**	**0.37 [0.18, 0.62]**	**-0.14 [-0.25, 0.00]**	**-0.16 [-0.27, -0.01]**	-0.04 [-0.11, 0.03]	-0.04 [-0.12, 0.03]	-0.05 [-0.13, 0.04]	-0.03 [-0.12, 0.05]
Total cholesterol, mmol/L	**0.48 [0.17, 0.84]**	0.32 [-0.01, 0.68]	**0.35 [0.12, 0.57]**	**0.32 [0.08, 0.56]**	0.11 [-0.15, 0.37]	0.09 [-0.18, 0.37]	0.02 [-0.10, 0.13]	0.01 [-0.12, 0.13]	-0.06 [-0.21, 0.08]	-0.08 [-0.24, 0.07]
HDL, mmol/L	**-0.30 [-0.40, -0.19]**	**-0.31 [-0.42, -0.19]**	**-0.32 [-0.39, -0.25]**	**-0.31 [-0.39, -0.23]**	0.09 [-0.04, 0.22]	0.11 [-0.03, 0.24]	-0.01 [-0.07, 0.05]	-0.01 [-0.08, 0.05]	0.02 [-0.06, 0.09]	0.03 [-0.05, 0.11]
LDL, mmol/L	**0.36 [0.08, 0.66]**	0.22 [-0.09, 0.53]	**0.38 [0.19, 0.59]**	**0.38 [0.16, 0.61]**	0.11 [-0.13, 0.34]	0.08 [-0.17, 0.33]	0.04 [-0.07, 0.15]	0.03 [-0.09, 0.15]	-0.06 [-0.19, 0.08]	-0.11 [-0.24, 0.04]
LDL/HDL ratio	**0.56 [0.25, 0.89]**	**0.44 [0.13, 0.79]**	**0.67 [0.45, 0.92]**	**0.67 [0.43, 0.95]**	0.00 [-0.19, 0.23]	-0.03 [-0.23, 0.21]	0.05 [-0.05, 0.15]	0.04 [-0.06, 0.16]	-0.05 [-0.17, 0.07]	-0.09 [-0.21, 0.04]
Triglyceride/HDL ratio	**0.58 [0.31, 0.95]**	**0.57 [0.28, 0.98]**	**0.49 [0.30, 0.72]**	**0.45 [0.26, 0.70]**	**-0.11 [-0.20, -0.01]**	**-0.13 [-0.22, -0.02]**	-0.02 [-0.07, 0.03]	-0.02 [-0.08, 0.04]	-0.04 [-0.10, 0.03]	-0.03 [-0.10, 0.05]
Leukocyte at 24 years, 10^9/L	**1.27 [0.53, 2.12]**	**1.17 [0.37, 2.08]**	**0.92 [0.40, 1.48]**	**0.89 [0.33, 1.51]**	-0.21 [-0.71, 0.35]	-0.25 [-0.79, 0.35]	**0.32 [0.05, 0.59]**	**0.39 [0.10, 0.69]**	0.04 [-0.27, 0.38]	-0.02 [-0.35, 0.34]
HbA1c at 26 years										
HbA1c, mmol/mol	**3.14 [1.54, 4.98]**	**2.94 [0.96, 5.20]**	0.05 [-1.28, 1.39]	0.53 [-0.91, 2.03]	0.34 [-0.80, 1.50]	-0.20 [-1.50, 1.11]	0.09 [-0.49, 0.66]	0.25 [-0.36, 0.88]	0.12 [-0.65, 0.89]	0.16 [-0.63, 0.95]

BP=blood pressure; HDL=high-density lipoprotein; LDL=low-density lipoprotein. HbA1c= Hemoglobin A1C.
Model 1 was adjusted for age.
Model 2 was additionally adjusted for smoking status, parental education, maternal smoking during pregnancy, maternal body mass index at early pregnancy, maternal hypertension, parity before the index person was born, and caesarean section.

Table S13. Mean differences and 95% confidence intervals for the associations of body mass index trajectories with cardiometabolic profile at late adolescence and young adulthood by linear regression in females. The stable normal group was the reference group.

	Increasing - persistent high		High - accelerated increasing		Increasing - accelerated resolving		Normal - above normal		Decreasing - persistent low	
Models	1	2	1	2	1	2	1	2	1	2
BP at 24 years										
Systolic BP, mmHg	4.99 [-0.46, 10.32]	3.03 [-2.61, 8.98]	**3.52 [0.91, 6.02]**	1.95 [-0.80, 4.88]	2.28 [-1.71, 6.33]	3.00 [-1.60, 7.64]	-0.62 [-2.22, 1.00]	-1.07 [-2.70, 0.76]	-1.53 [-3.05, 0.27]	-1.60 [-3.27, 0.37]
Diastolic BP, mmHg	**4.92 [1.16, 8.82]**	**4.84 [0.60, 9.15]**	1.29 [-0.81, 3.10]	0.68 [-1.50, 2.75]	1.01 [-2.12, 3.96]	2.12 [-1.47, 5.40]	-0.39 [-1.62, 1.06]	-0.47 [-1.81, 1.11]	-0.90 [-2.19, 0.64]	-0.85 [-2.26, 0.83]
Blood lipid at 24 years										
Triglyceride, mmol/L	**0.29 [0.04, 0.66]**	0.25 [-0.01, 0.65]	**0.33 [0.20, 0.49]**	**0.30 [0.16, 0.47]**	-0.13 [-0.24, 0.02]	-0.12 [-0.24, 0.05]	0.00 [-0.07, 0.05]	0.01 [-0.07, 0.06]	0.04 [-0.03, 0.10]	0.05 [-0.03, 0.12]
Total cholesterol, mmol/L	0.32 [-0.06, 0.74]	0.20 [-0.21, 0.65]	0.16 [-0.01, 0.33]	0.05 [-0.13, 0.24]	-0.10 [-0.37, 0.17]	-0.16 [-0.45, 0.14]	0.07 [-0.04, 0.19]	0.08 [-0.04, 0.21]	0.08 [-0.04, 0.21]	0.06 [-0.07, 0.20]
HDL, mmol/L	**-0.28 [-0.42, -0.12]**	**-0.28 [-0.44, -0.11]**	**-0.32 [-0.38, -0.26]**	**-0.32 [-0.39, -0.25]**	**-0.15 [-0.27, -0.01]**	**-0.19 [-0.31, -0.03]**	0.04 [-0.03, 0.10]	0.03 [-0.04, 0.10]	-0.02 [-0.09, 0.04]	-0.04 [-0.12, 0.03]
LDL, mmol/L	**0.48 [0.12, 0.91]**	0.37 [-0.02, 0.84]	**0.36 [0.21, 0.52]**	**0.27 [0.10, 0.44]**	0.11 [-0.14, 0.36]	0.09 [-0.19, 0.37]	0.06 [-0.05, 0.17]	0.07 [-0.05, 0.19]	0.10 [-0.02, 0.22]	0.09 [-0.03, 0.22]
LDL/HDL ratio	**0.60 [0.23, 1.04]**	**0.52 [0.12, 0.99]**	**0.64 [0.48, 0.83]**	**0.58 [0.39, 0.75]**	0.20 [-0.03, 0.46]	0.22 [-0.03, 0.52]	0.01 [-0.08, 0.11]	0.03 [-0.07, 0.13]	0.09 [-0.02, 0.20]	0.10 [-0.01, 0.22]
Triglyceride/HDL ratio	**0.37 [0.12, 0.73]**	**0.34 [0.07, 0.73]**	**0.45 [0.31, 0.61]**	**0.42 [0.28, 0.60]**	-0.03 [-0.13, 0.10]	0.00 [-0.12, 0.15]	-0.02 [-0.06, 0.04]	-0.01 [-0.06, 0.05]	0.04 [-0.02, 0.10]	0.05 [-0.01, 0.12]
Leukocyte at 24 years, 10^9/L	0.86 [-0.03, 1.91]	0.46 [-0.43, 1.53]	**0.81 [0.41, 1.24]**	**0.74 [0.32, 1.20]**	-0.45 [-0.94, 0.11]	-0.23 [-0.81, 0.42]	0.14 [-0.10, 0.40]	0.16 [-0.10, 0.44]	-0.05 [-0.31, 0.22]	-0.04 [-0.31, 0.25]
HbA1c at 26 years										
HbA1c, mmol/mol	**3.04 [1.05, 5.37]**	**4.24 [1.90, 7.48]**	**1.26 [0.45, 2.10]**	**1.44 [0.50, 2.42]**	-0.64 [-2.21, 0.96]	-0.59 [-2.42, 1.28]	-0.01 [-0.53, 0.52]	0.07 [-0.53, 0.66]	0.19 [-0.34, 0.72]	0.03 [-0.55, 0.62]

BP=blood pressure; HDL=high-density lipoprotein; LDL=low-density lipoprotein. HbA1c= Hemoglobin A1C.

Model 1 was adjusted for age.

Model 2 was additionally adjusted for smoking status, parental education, maternal smoking during pregnancy, maternal body mass index at early pregnancy, maternal hypertension, parity before the index person was born, and caesarean section.

Table S14. Mean differences and 95% confidence intervals of the sensitivity analysis which additionally included the BMI z scores at 24 years. The stable normal group was the reference group.

	Increasing - persistent high	High - accelerated increasing	Increasing - accelerated resolving	Normal - above normal	Decreasing - persistent low
BP at 24 years					
Systolic BP, mmHg	**-5.56 [-9.50, -1.86]**	**-3.15 [-5.59, -0.79]**	-0.57 [-3.28, 2.43]	-0.81 [-2.08, 0.49]	-0.44 [-1.98, 1.03]
Diastolic BP, mmHg	2.78 [-0.63, 5.87]	-0.06 [-2.06, 2.13]	0.15 [-2.13, 2.58]	-0.60 [-1.58, 0.54]	-0.01 [-1.23, 1.34]
Blood lipid at 24 years					
Triglyceride, mmol/L	0.04 [-0.12, 0.24]	0.02 [-0.09, 0.12]	**-0.20 [-0.27, -0.11]**	-0.05 [-0.10, 0.00]	0.05 [0.00, 0.12]
Total cholesterol, mmol/L	-0.05 [-0.33, 0.25]	-0.10 [-0.28, 0.08]	-0.09 [-0.30, 0.11]	0.01 [-0.08, 0.10]	0.06 [-0.04, 0.16]
HDL, mmol/L	-0.01 [-0.16, 0.14]	**-0.10 [-0.19, -0.01]**	0.06 [-0.05, 0.16]	0.05 [0.00, 0.09]	**-0.07 [-0.12, -0.01]**
LDL, mmol/L	-0.18 [-0.42, 0.08]	-0.06 [-0.22, 0.11]	-0.03 [-0.21, 0.16]	0.00 [-0.08, 0.09]	0.09 [-0.01, 0.19]
LDL/HDL ratio	-0.16 [-0.34, 0.06]	0.06 [-0.09, 0.22]	-0.07 [-0.22, 0.09]	-0.03 [-0.10, 0.04]	**0.12 [0.03, 0.21]**
Triglyceride/HDL ratio	0.02 [-0.10, 0.18]	0.05 [-0.03, 0.15]	**-0.14 [-0.20, -0.07]**	**-0.05 [-0.08, -0.01]**	**0.07 [0.02, 0.13]**
Leukocyte at 24 years, 10^9/L	-0.09 [-0.67, 0.56]	-0.02 [-0.39, 0.39]	**-0.50 [-0.88, -0.09]**	0.15 [-0.04, 0.35]	0.11 [-0.11, 0.35]
HbA1c at 26 years					
HbA1c, mmol/mol	**3.00 [1.34, 4.82]**	0.66 [-0.31, 1.65]	-0.39 [-1.47, 0.69]	0.06 [-0.37, 0.49]	0.13 [-0.35, 0.60]

BP=blood pressure; HDL=high-density lipoprotein; LDL=low-density lipoprotein. HbA1c= Hemoglobin A1C.

Models were adjusted for age, sex, BIM z scores at birth, smoking status, parental education, maternal smoking during pregnancy, maternal body mass index at early pregnancy, maternal hypertension, parity before the index person was born, and caesarean section.

Table S15. Mean differences and 95% confidence intervals of the sensitivity analysis which additionally included the BMI z scores at birth. The stable normal group was the reference group.

	Increasing - persistent high	High - accelerated increasing	Increasing - accelerated resolving	Normal - above normal	Decreasing - persistent low
BP at 24 years					
Systolic BP, mmHg	3.01 [-0.32, 6.37]	**3.44 [1.41, 5.51]**	1.54 [-1.16, 4.40]	0.23 [-0.95, 1.45]	**-1.67 [-2.82, -0.27]**
Diastolic BP, mmHg	**4.67 [2.29, 7.03]**	**2.16 [0.67, 3.62]**	0.78 [-1.43, 2.83]	-0.39 [-1.33, 0.66]	-0.38 [-1.44, 0.84]
Blood lipid at 24 years					
Triglyceride, mmol/L	**0.49 [0.27, 0.75]**	**0.35 [0.24, 0.48]**	**-0.14 [-0.21, -0.04]**	-0.02 [-0.07, 0.03]	0.00 [-0.05, 0.05]
Total cholesterol, mmol/L	**0.42 [0.18, 0.69]**	**0.23 [0.09, 0.36]**	0.01 [-0.17, 0.20]	0.05 [-0.03, 0.13]	0.02 [-0.07, 0.11]
HDL, mmol/L	**-0.30 [-0.38, -0.21]**	**-0.32 [-0.37, -0.27]**	-0.02 [-0.12, 0.07]	0.01 [-0.03, 0.05]	-0.01 [-0.06, 0.04]
LDL, mmol/L	**0.41 [0.19, 0.65]**	**0.36 [0.24, 0.49]**	0.11 [-0.07, 0.28]	0.05 [-0.02, 0.13]	0.03 [-0.06, 0.12]
LDL/HDL ratio	**0.58 [0.34, 0.84]**	**0.65 [0.52, 0.79]**	0.09 [-0.06, 0.26]	0.03 [-0.03, 0.10]	0.03 [-0.05, 0.11]
Triglyceride/HDL ratio	**0.51 [0.31, 0.77]**	**0.46 [0.34, 0.58]**	-0.08 [-0.15, 0.00]	-0.02 [-0.05, 0.02]	0.00 [-0.04, 0.05]
Leukocyte at 24 years, 10^9/L	**1.11 [0.53, 1.75]**	**0.85 [0.53, 1.19]**	-0.33 [-0.69, 0.05]	**0.23 [0.05, 0.41]**	-0.01 [-0.21, 0.19]
HbA1c at 26 years					
HbA1c, mmol/mol	**3.10 [1.86, 4.41]**	**0.94 [0.25, 1.64]**	0.04 [-0.89, 0.97]	0.04 [-0.35, 0.43]	0.16 [-0.27, 0.60]

BP=blood pressure; HDL=high-density lipoprotein; LDL=low-density lipoprotein. HbA1c= Hemoglobin A1C.

Models were adjusted for age, sex, BIM z scores at birth, smoking status, parental education, maternal smoking during pregnancy, maternal body mass index at early pregnancy, maternal hypertension, parity before the index person was born, and caesarean section.

Table S16. Blood cell counts at 24 years according to body mass index trajectories.

	Increasing - persistent high (n=39)	High - accelerated increasing (n=135)	Increasing - accelerated resolving (n=64)	Normal - above normal (n=442)	Stable normal (n=999)	Decreasing - persistent low (n=294)	p
Leukocyte, 10^9/L	7.38 ± 1.93	7.23 ± 1.76	5.89 ± 1.22	6.53 ± 1.66	6.36 ± 1.70	6.38 ± 1.80	< 0.001
Neutrophil, %	57.21 ± 7.38	55.42 ± 8.42	54.02 ± 9.14	56.92 ± 9.41	56.14 ± 9.24	56.12 ± 8.52	0.167
Lymphocyte, %	31.85 ± 6.98	33.13 ± 7.91	34.27 ± 8.28	32.12 ± 8.42	32.67 ± 8.21	32.53 ± 7.55	0.392
Monocytes, %	7.63 ± 1.47	7.85 ± 2.06	8.53 ± 2.33	7.90 ± 2.13	8.02 ± 2.17	8.05 ± 2.30	0.250
Eosinophil, %	2.05 ± 1.95	2.17 ± 2.24	2.02 ± 2.93	1.69 ± 1.94	1.82 ± 2.40	1.97 ± 2.60	0.284
Basophils, %	0.00 ± 0.00	0.00 ± 0.00	0.00 ± 0.00	0.02 ± 0.20	0.02 ± 0.14	0.02 ± 0.17	0.595

Table S17. Mean differences and 95% confidence intervals for the associations of body mass index trajectories with inflammation-related proteins in young adulthood by linear regression. The stable normal group was the reference group.

Proteins	Increasing - persistent high		High - accelerated increasing		Increasing - accelerated resolving		Normal - above normal		Decreasing - persistent low	
	β [95% CI]	p*	β [95% CI]	p*	β [95% CI]	p*	β [95% CI]	p*	β [95% CI]	p*
**CDCP1**	**1.08 [0.72, 1.44]**	**<0.001**	**0.78 [0.58, 0.99]**	**<0.001**	-0.05 [-0.32, 0.22]	0.852	-0.01 [-0.13, 0.11]	0.942	-0.21 [-0.35, -0.07]	0.012
**MCP3**	**1.19 [0.82, 1.56]**	**<0.001**	**0.83 [0.63, 1.03]**	**<0.001**	0.23 [-0.05, 0.50]	0.260	0.00 [-0.12, 0.12]	0.987	-0.11 [-0.26, 0.03]	0.285
**IL6**	**0.90 [0.52, 1.28]**	**<0.001**	**0.73 [0.52, 0.93]**	**<0.001**	0.23 [-0.05, 0.52]	0.263	-0.03 [-0.15, 0.10]	0.833	-0.06 [-0.20, 0.09]	0.659
**IL18R1**	**0.89 [0.53, 1.25]**	**<0.001**	**0.65 [0.45, 0.86]**	**<0.001**	-0.03 [-0.31, 0.24]	0.898	-0.05 [-0.18, 0.07]	0.610	-0.06 [-0.20, 0.08]	0.656
**HGF**	**0.92 [0.58, 1.26]**	**<0.001**	**0.70 [0.51, 0.89]**	**<0.001**	0.10 [-0.16, 0.36]	0.669	-0.03 [-0.15, 0.09]	0.810	-0.01 [-0.14, 0.13]	0.964
**VEGFA**	**0.80 [0.44, 1.16]**	**<0.001**	**0.58 [0.38, 0.78]**	**<0.001**	-0.16 [-0.43, 0.11]	0.465	-0.03 [-0.16, 0.09]	0.782	-0.05 [-0.19, 0.09]	0.671
**CCL3**	**0.68 [0.31, 1.05]**	**<0.001**	**0.65 [0.44, 0.86]**	**<0.001**	0.17 [-0.11, 0.46]	0.440	0.02 [-0.10, 0.15]	0.865	-0.08 [-0.22, 0.06]	0.480
**FGF21**	**0.70 [0.33, 1.07]**	**<0.001**	**0.42 [0.21, 0.63]**	**<0.001**	0.00 [-0.29, 0.28]	0.987	-0.19 [-0.31, -0.07]	0.012	-0.01 [-0.15, 0.13]	0.932
TRAIL	**0.55 [0.20, 0.90]**	**0.009**	**0.45 [0.26, 0.64]**	**<0.001**	**0.54 [0.28, 0.81]**	**<0.001**	0.07 [-0.04, 0.19]	0.433	-0.09 [-0.22, 0.05]	0.422
CCL4	**0.48 [0.12, 0.85]**	**0.035**	**0.61 [0.41, 0.81]**	**<0.001**	-0.20 [-0.47, 0.08]	0.351	0.02 [-0.11, 0.14]	0.896	0.06 [-0.08, 0.20]	0.637
IL18	**0.67 [0.31, 1.03]**	**<0.001**	**0.41 [0.21, 0.61]**	**<0.001**	-0.04 [-0.31, 0.23]	0.892	-0.04 [-0.17, 0.08]	0.705	-0.21 [-0.35, -0.07]	0.012
TNFSF14	**0.75 [0.40, 1.11]**	**<0.001**	**0.49 [0.30, 0.69]**	**<0.001**	-0.05 [-0.32, 0.22]	0.836	0.00 [-0.12, 0.12]	0.987	-0.01 [-0.15, 0.12]	0.929
FGF23	0.42 [0.04, 0.80]	0.089	**0.55 [0.34, 0.75]**	**<0.001**	0.09 [-0.20, 0.38]	0.744	-0.06 [-0.19, 0.06]	0.567	-0.08 [-0.23, 0.07]	0.518
MCP4	**0.85 [0.49, 1.21]**	**<0.001**	**0.47 [0.27, 0.67]**	**<0.001**	0.22 [-0.06, 0.49]	0.277	-0.01 [-0.13, 0.12]	0.960	0.09 [-0.04, 0.23]	0.387
CCL19	**0.59 [0.21, 0.98]**	**0.012**	**0.36 [0.15, 0.57]**	**0.005**	0.06 [-0.23, 0.35]	0.832	-0.12 [-0.24, 0.01]	0.203	0.01 [-0.13, 0.16]	0.922
MCP1	**0.72 [0.35, 1.08]**	**<0.001**	**0.46 [0.26, 0.66]**	**<0.001**	0.05 [-0.23, 0.33]	0.851	-0.03 [-0.15, 0.09]	0.831	0.04 [-0.10, 0.19]	0.744
LAPTGFbeta1	**0.76 [0.39, 1.12]**	**<0.001**	**0.37 [0.17, 0.58]**	**<0.001**	-0.06 [-0.33, 0.22]	0.836	-0.08 [-0.20, 0.04]	0.413	-0.05 [-0.19, 0.10]	0.725
IL10RB	**0.69 [0.32, 1.07]**	**<0.001**	**0.37 [0.16, 0.57]**	**0.005**	0.04 [-0.25, 0.32]	0.898	-0.11 [-0.23, 0.02]	0.240	0.05 [-0.10, 0.19]	0.729
TRANCE	0.38 [0.02, 0.75]	0.122	**0.37 [0.17, 0.57]**	**<0.001**	0.33 [0.06, 0.61]	0.059	0.05 [-0.07, 0.17]	0.621	-0.02 [-0.16, 0.12]	0.896
AXIN1	**0.72 [0.37, 1.07]**	**<0.001**	**0.37 [0.17, 0.56]**	**<0.001**	0.00 [-0.26, 0.27]	0.987	-0.06 [-0.18, 0.06]	0.557	-0.05 [-0.18, 0.09]	0.710
OSM	**0.63 [0.25, 1.00]**	**0.005**	**0.39 [0.19, 0.60]**	**<0.001**	-0.20 [-0.48, 0.08]	0.369	0.07 [-0.06, 0.19]	0.507	0.04 [-0.11, 0.18]	0.799
SIRT2	**0.67 [0.30, 1.03]**	**<0.001**	**0.35 [0.15, 0.55]**	**0.005**	-0.09 [-0.37, 0.18]	0.712	-0.08 [-0.20, 0.05]	0.432	-0.05 [-0.19, 0.09]	0.690
STAMBP	**0.67 [0.32, 1.03]**	**<0.001**	**0.35 [0.15, 0.54]**	**0.005**	-0.06 [-0.33, 0.21]	0.831	-0.07 [-0.19, 0.05]	0.452	-0.03 [-0.16, 0.11]	0.836
CD40	**0.67 [0.31, 1.03]**	**<0.001**	**0.31 [0.10, 0.51]**	**0.012**	0.06 [-0.22, 0.33]	0.836	-0.04 [-0.16, 0.08]	0.741	-0.06 [-0.20, 0.08]	0.637
Flt3L	0.24 [-0.13, 0.60]	0.413	0.17 [-0.04, 0.37]	0.258	0.01 [-0.27, 0.28]	0.970	**-0.16 [-0.28, -0.04]**	**0.032**	0.08 [-0.06, 0.23]	0.462
ADA	**0.47 [0.11, 0.83]**	**0.038**	**0.35 [0.15, 0.55]**	**0.005**	0.02 [-0.25, 0.30]	0.929	-0.03 [-0.15, 0.09]	0.818	-0.12 [-0.26, 0.02]	0.220
IL22RA1	**0.60 [0.22, 0.99]**	**0.009**	**0.37 [0.16, 0.59]**	**0.005**	-0.03 [-0.32, 0.26]	0.905	0.00 [-0.13, 0.13]	0.987	0.02 [-0.13, 0.17]	0.885
4EBP1	**0.56 [0.21, 0.92]**	**0.009**	**0.35 [0.15, 0.55]**	**<0.001**	-0.12 [-0.39, 0.15]	0.597	-0.06 [-0.18, 0.06]	0.571	0.01 [-0.13, 0.14]	0.964
IL7	**0.78 [0.41, 1.15]**	**<0.001**	**0.36 [0.16, 0.56]**	**0.005**	-0.13 [-0.41, 0.15]	0.591	0.02 [-0.10, 0.14]	0.870	-0.07 [-0.21, 0.08]	0.575
CSF1	0.38 [0.02, 0.74]	0.112	**0.31 [0.11, 0.51]**	**0.009**	-0.02 [-0.29, 0.25]	0.929	-0.01 [-0.13, 0.12]	0.964	-0.06 [-0.20, 0.08]	0.597
IL12B	0.31 [-0.06, 0.68]	0.240	**0.38 [0.17, 0.58]**	**<0.001**	-0.02 [-0.29, 0.26]	0.949	-0.04 [-0.16, 0.09]	0.744	-0.05 [-0.19, 0.08]	0.660
IL8	**0.61 [0.24, 0.98]**	**0.005**	**0.44 [0.24, 0.65]**	**<0.001**	-0.09 [-0.37, 0.19]	0.725	-0.07 [-0.20, 0.05]	0.479	0.03 [-0.11, 0.17]	0.831

CXCL5	**0.55 [0.18, 0.93]**	**0.016**	**0.29 [0.08, 0.49]**	**0.022**	0.08 [-0.20, 0.36]	0.744	0.02 [-0.11, 0.14]	0.898	-0.07 [-0.21, 0.07]	0.557
ENRAGE	0.35 [-0.02, 0.72]	0.172	0.25 [0.05, 0.45]	0.053	0.05 [-0.23, 0.32]	0.851	0.10 [-0.03, 0.22]	0.279	-0.06 [-0.20, 0.08]	0.591
CCL20	0.25 [-0.12, 0.62]	0.392	0.20 [-0.01, 0.40]	0.157	0.10 [-0.18, 0.38]	0.690	**-0.18 [-0.30, -0.05]**	**0.022**	-0.01 [-0.16, 0.13]	0.929
CXCL6	**0.52 [0.15, 0.88]**	**0.022**	**0.35 [0.14, 0.55]**	**0.005**	-0.01 [-0.29, 0.26]	0.964	0.02 [-0.10, 0.15]	0.836	-0.04 [-0.18, 0.10]	0.758
CD5	0.41 [0.05, 0.77]	0.085	**0.41 [0.21, 0.61]**	**<0.001**	0.05 [-0.22, 0.32]	0.836	-0.02 [-0.14, 0.11]	0.898	-0.05 [-0.19, 0.10]	0.725
SCF	-0.21 [-0.58, 0.16]	0.479	-0.22 [-0.43, -0.01]	0.112	0.10 [-0.17, 0.37]	0.683	0.05 [-0.07, 0.17]	0.640	0.08 [-0.07, 0.22]	0.521
IL10	0.26 [-0.12, 0.64]	0.379	0.11 [-0.09, 0.32]	0.486	0.01 [-0.27, 0.30]	0.964	-0.07 [-0.20, 0.06]	0.482	**-0.21 [-0.35, -0.07]**	**0.016**
IL2	0.35 [-0.03, 0.73]	0.184	**0.29 [0.08, 0.50]**	**0.022**	-0.29 [-0.58, 0.00]	0.135	-0.04 [-0.16, 0.09]	0.744	-0.10 [-0.24, 0.05]	0.386
CASP8	0.39 [0.05, 0.73]	0.080	0.22 [0.03, 0.40]	0.078	-0.25 [-0.50, 0.01]	0.162	0.01 [-0.10, 0.13]	0.908	-0.04 [-0.18, 0.09]	0.722
ST1A1	**0.54 [0.17, 0.90]**	**0.016**	0.20 [0.00, 0.40]	0.131	-0.15 [-0.42, 0.12]	0.486	-0.08 [-0.20, 0.04]	0.386	-0.07 [-0.21, 0.07]	0.571
CXCL10	0.36 [-0.03, 0.75]	0.181	**0.27 [0.06, 0.48]**	**0.044**	0.17 [-0.12, 0.46]	0.479	0.02 [-0.11, 0.15]	0.892	-0.09 [-0.23, 0.06]	0.471
IL20	0.28 [-0.11, 0.67]	0.345	**0.33 [0.12, 0.55]**	**0.009**	-0.18 [-0.47, 0.11]	0.446	0.10 [-0.03, 0.23]	0.281	0.03 [-0.11, 0.18]	0.824
MCP2	**0.49 [0.12, 0.86]**	**0.032**	0.13 [-0.07, 0.34]	0.413	0.09 [-0.18, 0.37]	0.712	0.00 [-0.12, 0.13]	0.987	-0.08 [-0.22, 0.06]	0.486
IL5	0.18 [-0.20, 0.57]	0.571	**0.27 [0.06, 0.48]**	**0.044**	0.15 [-0.14, 0.43]	0.527	0.03 [-0.10, 0.16]	0.831	-0.09 [-0.23, 0.06]	0.452
CXCL1	**0.65 [0.27, 1.03]**	**0.005**	0.23 [0.02, 0.44]	0.094	0.09 [-0.20, 0.37]	0.742	0.03 [-0.10, 0.15]	0.836	-0.09 [-0.23, 0.06]	0.452
SLAMF1	0.33 [-0.05, 0.70]	0.219	0.20 [0.00, 0.41]	0.156	0.23 [-0.05, 0.50]	0.265	0.02 [-0.11, 0.14]	0.892	-0.14 [-0.28, 0.01]	0.162
IL33	0.40 [0.02, 0.77]	0.111	**0.34 [0.13, 0.54]**	**0.005**	-0.04 [-0.32, 0.24]	0.890	0.04 [-0.09, 0.16]	0.744	0.12 [-0.03, 0.26]	0.265
IL17A	0.08 [-0.32, 0.47]	0.836	0.08 [-0.14, 0.30]	0.670	-0.09 [-0.39, 0.20]	0.736	-0.14 [-0.27, -0.02]	0.089	-0.04 [-0.19, 0.11]	0.782
CCL28	-0.08 [-0.46, 0.30]	0.832	-0.21 [-0.42, -0.01]	0.124	0.02 [-0.27, 0.30]	0.963	0.06 [-0.07, 0.18]	0.597	0.09 [-0.06, 0.24]	0.452
TNFRSF9	0.26 [-0.10, 0.62]	0.357	0.19 [-0.01, 0.40]	0.163	0.01 [-0.27, 0.28]	0.970	-0.11 [-0.23, 0.02]	0.235	-0.06 [-0.19, 0.08]	0.656
PDL1	0.28 [-0.07, 0.64]	0.279	0.24 [0.05, 0.44]	0.053	0.13 [-0.14, 0.40]	0.575	-0.08 [-0.20, 0.04]	0.413	-0.08 [-0.22, 0.06]	0.482

* FDR adjusted p values.

Models were adjusted for smoking status, parental education, maternal smoking during pregnancy, maternal body mass index at early pregnancy, maternal hypertension, parity before the index person was born, and caesarean section.

Table S18. The list of studies included in the literature review.

Study; Country	Title
**Birth cohort**	
Kim et al, 2023; South Korea	BMI trajectory and inflammatory effects on metabolic syndrome in adolescents
Perez et al, 2023; USA	Association of BMI trajectories with cardiometabolic risk among low-income Mexican American children
Montazeri et al, 2022; Spain	Early-childhood BMI trajectories in relation to preclinical cardiovascular measurements in adolescence
Ford et al, 2020; Guatemala	Lifecourse body mass index trajectories and cardio-metabolic disease risk in Guatemalan adults
Liu et al, 2020; Australia	Do body mass index and waist-to-height ratio over the preceding decade predict retinal microvasculature in 11–12 year olds and midlife adults?
Barraclough et al, 2019; Australia	Weight Gain Trajectories from Birth to Adolescence and Cardiometabolic Status in Adolescence
Huang et al, 2019; Australia	Lifecourse Childhood Adiposity Trajectories Associated With Adolescent Insulin Resistance
Li et al, 2019; Canada	The association between body mass index trajectories and cardiometabolic risk in young children
Wibaek et al, 2019; Ethiopia	Body mass index trajectories in early childhood in relation to cardiometabolic risk profile and body composition at 5 years of age
Aris et al, 2017; Singapore	Body mass index trajectories in the first two years and subsequent childhood cardio-metabolic outcomes: a prospective multiethnic Asian cohort study
Hanvey et al, 2017; Australia	Adolescent Cardiovascular Functional and Structural Outcomes of Growth Trajectories from Infancy: Prospective Community-Based Study
**Others**	
Brito et al, 2023; USA	Body Mass Index Trajectories and Biomarkers of Cardiometabolic Risk in Children from Low-Income and Racially and Ethnically Diverse Households
Wang et al, 2023; China	Early-Life Cardiovascular Risk Factor Trajectories and Vascular Aging in Midlife: A 30-Year Prospective Cohort Study
Blond et al, 2022; Denmark	Body mass index trajectories in childhood and incidence rates of type 2 diabetes and coronary heart disease in adulthood: A cohort study
Ge et al, 2022; China	Pediatric body mass index trajectories and the risk of hypertension among adolescents in China: a retrospective cohort study
Wang et al, 2022a; China	Early life body mass index trajectories and albuminuria in midlife: A 30-year prospective cohort study
Wang et al, 2022b; China	Exploring Overweight Risk Trajectories During Childhood and Their Associations With Elevated Blood Pressure at Late Adolescence: a Retrospective Cohort Study
Beales et al, 2021; Australia	Insight into the longitudinal relationship between chronic subclinical inflammation and obesity from adolescence to early adulthood: a dual trajectory analysis
Ji et al, 2021; China	Body mass index trajectory from childhood to puberty and high blood pressure: the China Health and Nutrition Survey
Norris et al, 2021; UK	Distinct Body Mass Index Trajectories to Young-Adulthood Obesity and Their Different Cardiometabolic Consequences
Teng et al, 2021; China	Body Mass Index Trajectories during 6–18 Years Old and the Risk of Hypertension in Young Adult: A Longitudinal Study in Chinese Population
Wu et al, 2021; Taiwan	Adolescent Tri-ponderal Mass Index Growth Trajectories and Incident Diabetes Mellitus in Early Adulthood
Blond et al, 2020; Denmark	Associations between body mass index trajectories in childhood and cardiovascular risk factors in adulthood
Lycett et al, 2020; Australia	Body Mass Index From Early to Late Childhood and Cardiometabolic Measurements at 11 to 12 Years

Wang et al, 2020; China	Body Mass Index Trajectory and Incident Hypertension: Results From a Longitudinal Cohort of Chinese Children and Adolescents, 2006–2016
Yuan et al, 2020; China	Body Mass Index Trajectories in Early Life Is Predictive of Cardiometabolic Risk
Oluwagbemigun et al, 2019; Germany	Developmental trajectories of body mass index from childhood into late adolescence and subsequent late adolescence–young adulthood cardiometabolic risk markers
Buscot et al, 2018; Finland	Distinct child-to-adult body mass index trajectories are associated with different levels of adult cardiometabolic risk
Hao et al, 2018; USA	Body mass index trajectories in childhood is predictive of cardiovascular risk: results from the 23-year longitudinal Georgia Stress and Heart study
Araújo et al, 2016; Portugal	Trajectories of total and central adiposity throughout adolescence and cardiometabolic factors in early adulthood
Munthali et al, 2016; South Africa	Childhood adiposity trajectories are associated with late adolescent blood pressure: birth to twenty cohort
