# Supplementary material for: BMI trajectories from birth to young adulthood associate with distinct cardiometabolic profiles
Source: BMC Med. 2024 Nov 5;22:510. doi: 10.1186/s12916-024-03741-0 (PMC11539615; doi:10.1186/s12916-024-03741-0)
Supplement: Supplementary file 2 — Additional file 2: Figures S1–S7. Fig. S1 Flow chart of study participants in the BAMSE cohort. Fig. S2 Body mass index trajectories from birth to young adulthood in the BAMSE cohort. Fig. S3 Body mass index z-scores for participants with or without BMI data at 24 years of age in six BMI groups. Fig. S4 Body mass index values for participants with or without BMI data at 24 years of age in six BMI groups. Fig. S5 Association of body mass index trajectories with blood pressure, blood lipid, and HbA1c in young adulthood after additionally adjusting for fat mass index determined by linear regression. Fig. S6 Association of BMI trajectories with any heightened cardiometabolic risk at young adulthood determined by logistic regression. Fig. S7 Protein expression levels in the heart muscle, smooth muscle, and adipose tissue. [file 12916_2024_3741_MOESM2_ESM.pdf]

28 October 2024

Additional file 2

"BMI trajectories from birth to young adulthood associate with distinct cardiometabolic profiles"

Authors:

Gang Wang*#; Dang Wei#; Simon Kebede Merid; Sandra Ekström; Susanna Klevebro; Natalia Hernandez-Pacheco; Sophia Björkander; Petter Ljungman; Inger Kull; Jochen M Schwenk; Anna Bergström##; Erik Melén##

# Equal contributors of first authors.

Equal contributors of last authors.

* Corresponding Author

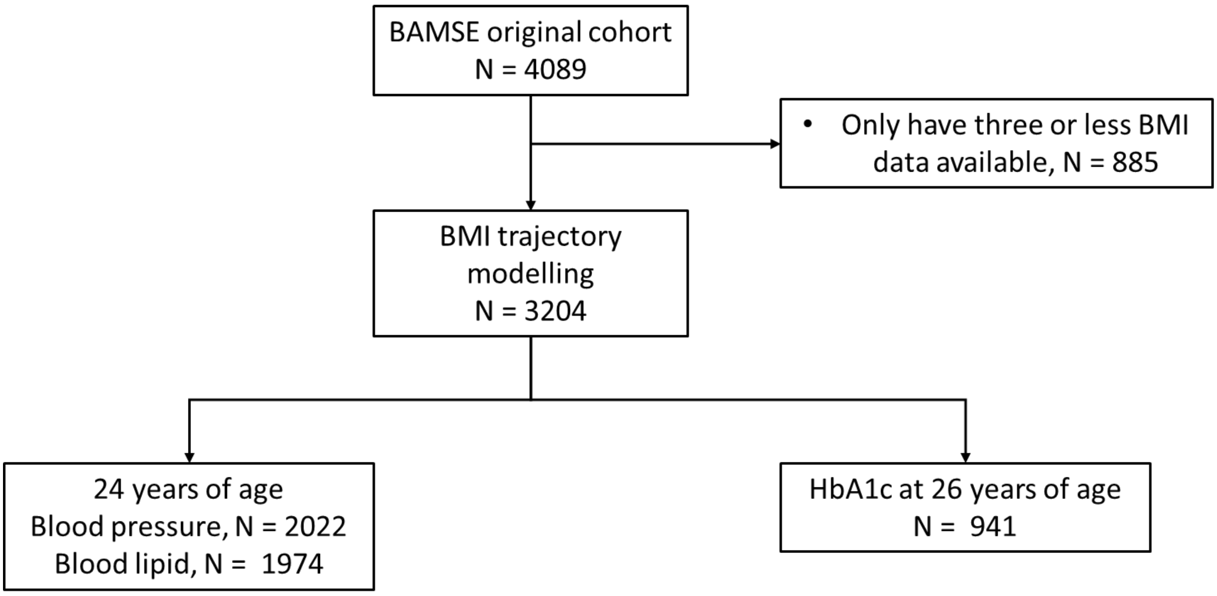

Figure S1. Flow chart of study participants in the BAMSE cohort.

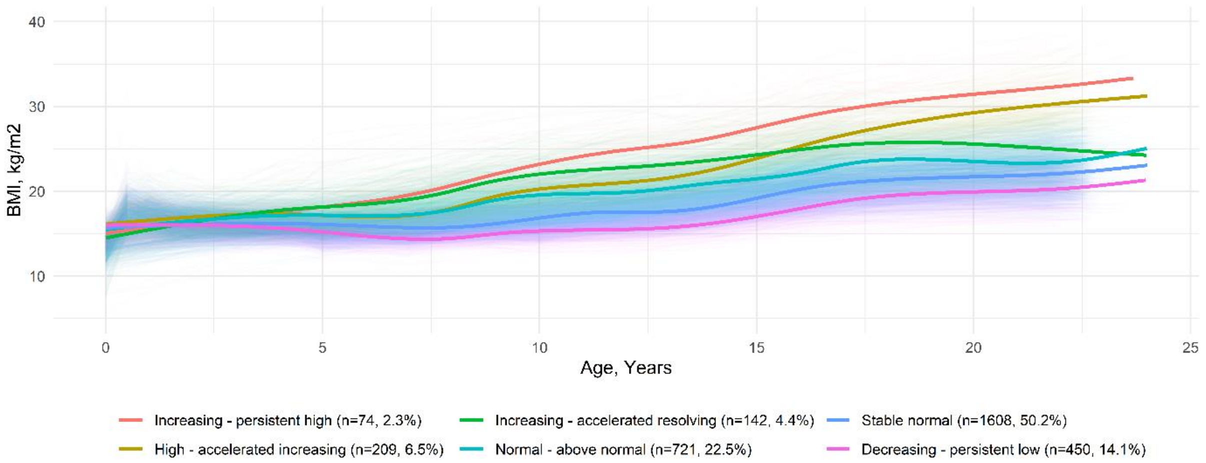

Figure S2. Body mass index trajectories from birth to young adulthood in the BAMSE cohort. Thin lines show the observed raw values of individual body mass index. Solid lines show the loess-smoothed body mass index trajectories for raw BMI values for the six identified trajectory groups.

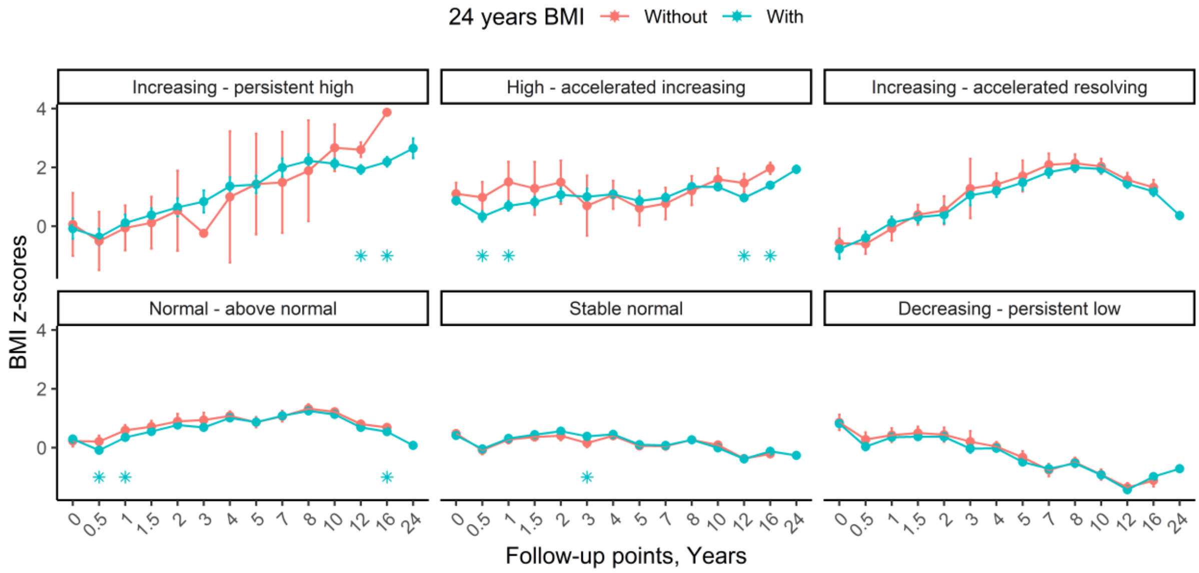

Figure S3. Body mass index z scores for participants with or without BMI data at 24 years of age in six BMI groups.

The results were illustrated with mean ± 95% confidence interval.

* $p<0.05$ for comparisons between participants with or without BMI data at 24 years.

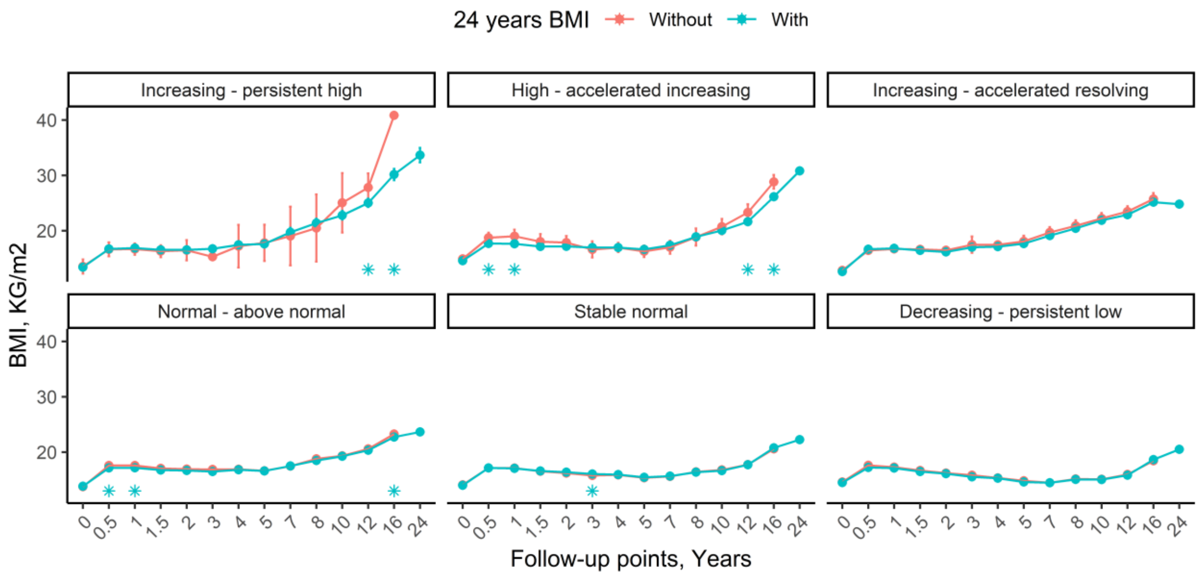

Figure S4. Body mass index values for participants with or without BMI data at 24 years of age in six BMI groups.

The results were illustrated with mean ± 95% confidence interval.

* p<0.05 for comparisons between participants with or without BMI data at 24 years.

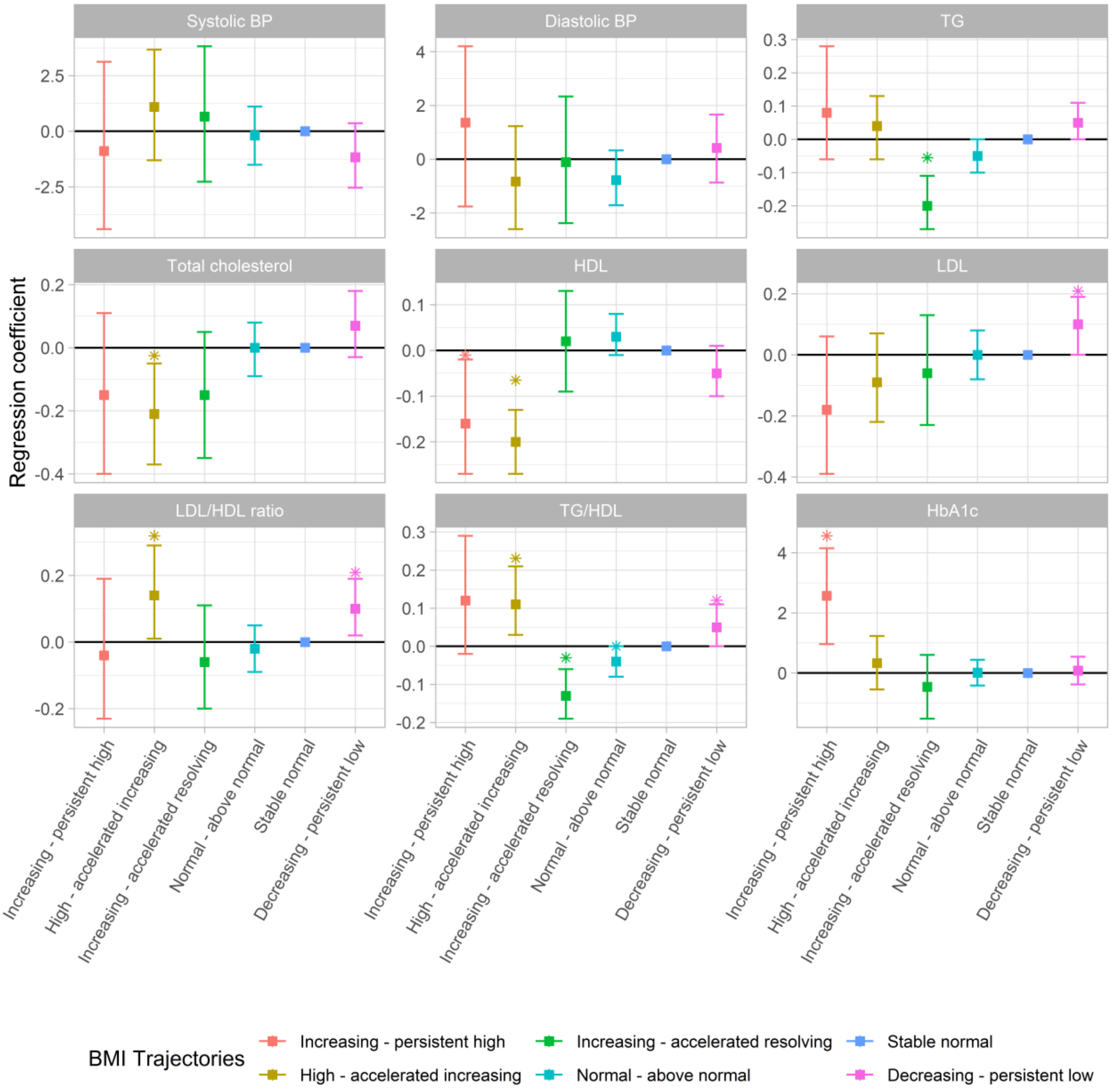

Figure S5. Association of body mass index trajectories with blood pressure, blood lipid, and HbA1c in young adulthood after additionally adjusting for fat mass index determined by linear regression. The stable normal group was the reference group. The y-axis displays the β coefficients along with their corresponding 95% confidence intervals. The models were adjusted for age, sex, smoking status, parental education, maternal smoking during pregnancy, maternal body mass index at early pregnancy, maternal hypertension, parity before the index person was born, cesarean section, and fat mass index at young adulthood. BP=blood pressure; TG=triglyceride; HDL=high density lipoprotein; LDL=low density lipoprotein; HbA1c=hemoglobin A1c.

*: Significant difference between Stable normal and other groups ($p<0.05$).

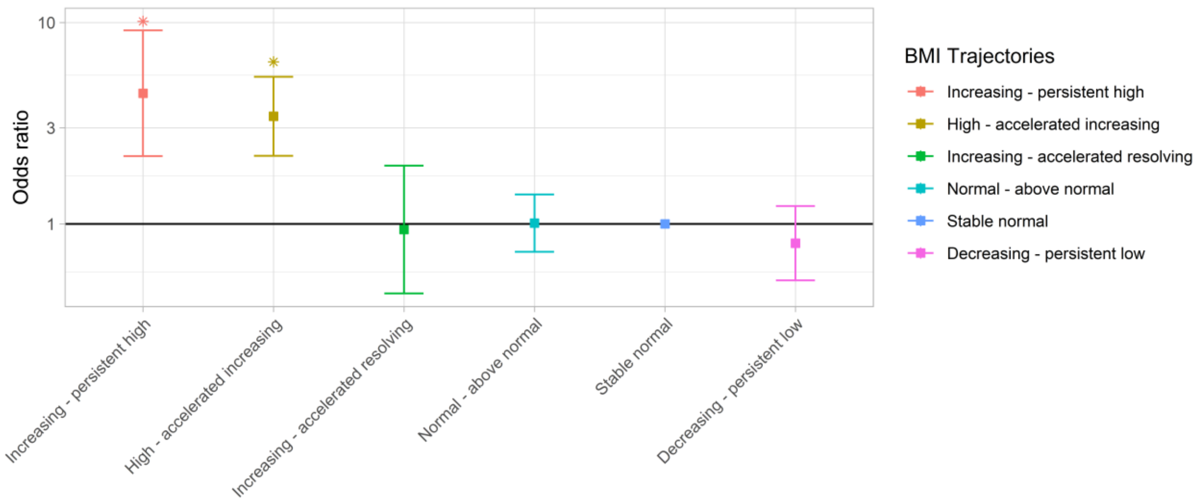

Figure S6. **Association of BMI trajectories with any heightened cardiometabolic risk at young adulthood determined by logistic regression.** The stable normal BMI group was the reference group. The models were adjusted for age, sex, smoking status, parental education, maternal smoking during pregnancy, maternal body mass index at early pregnancy, maternal hypertension, parity before the index person was born, and cesarean section.

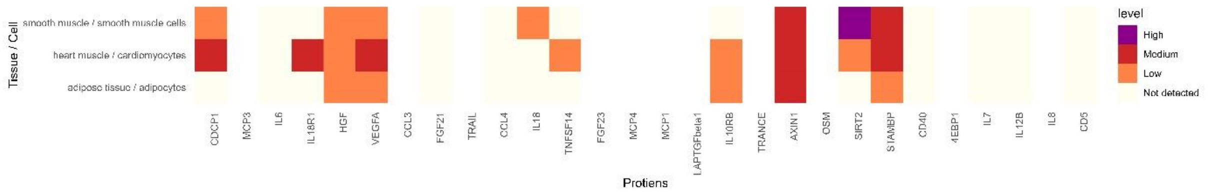

Figure S7. **Protein expression levels in the heart muscle, smooth muscle, and adipose tissue.** Only the 28 proteins showing increased levels in the persistent high and accelerated increasing BMI groups were included. Protein expression scores were derived from immunohistochemical data, manually assessed for staining intensity (negative, weak, moderate, or strong) and fraction of stained cells (<25%, 25-75%, or >75%). These combinations were then automatically converted into protein expression level scores: negative (not detected); weak <25% (not detected); weak combined with either 25-75% or >75% (low); moderate <25% (low); moderate combined with either 25-75% or >75% (medium); strong <25% (medium); strong combined with either 25-75% or >75% (high). Furthermore, protein expression values were manually adjusted by expert annotators in the Human Protein Atlas as needed.
