# Supplementary material for: BMI trajectories from birth to young adulthood associate with distinct cardiometabolic profiles
Source: BMC Med. 2024 Nov 5;22:510. doi: 10.1186/s12916-024-03741-0 (PMC11539615; doi:10.1186/s12916-024-03741-0)
Supplement: Supplementary file 3 — Additional file 3. Additional methods. [file 12916_2024_3741_MOESM3_ESM.pdf]

28 October 2024

Additional file 3

“BMI trajectories from birth to young adulthood associate with distinct cardiometabolic profiles”

Authors:

Gang Wang*#; Dang Wei#; Simon Kebede Merid; Sandra Ekström; Susanna Klevebro; Natalia Hernandez-Pacheco; Sophia Björkander; Petter Ljungman; Inger Kull; Jochen M Schwenk; Anna Bergström##; Erik Melén##

# Equal contributors of first authors.

Equal contributors of last authors.

* Corresponding Author

METHODS

Definitions of potential confounders and covariates

Preterm birth was defined as babies who were delivered before 37 completed weeks of gestation.

Early-life bronchitis, pneumonia, and respiratory syncytial virus infection were based on parental questionnaire data at the first-year follow-up.

Smoking status and snuff use were collected at 16- and 24-year follow-up questionnaires by participants themselves.

Maternal smoking during pregnancy was defined as the mother smoking at least one cigarette per day at any point in time during pregnancy.

Maternal height and weight were assessed during early pregnancy by midwives, typically between 8 and 12 weeks of gestation at the midwifery clinic in Sweden. Body mass index was computed as the ratio of weight in kilograms to the square of height in meters (kg/m^2).

Maternal hypertension during pregnancy was documented by midwives in the midwifery clinic or identified through the International Classification of Diseases, 10th Revision (ICD-10) codes collected in the Swedish Medical Birth Register (ICD-10 code: 642).

Maternal diabetes mellitus during pregnancy was documented by midwives in the midwifery clinic or identified through ICD-10 codes collected in the Swedish Medical Birth Register (ICD-10 code: 648A or 648W).

Participants were classified as having high-risk lipid levels if their low-density lipoprotein cholesterol levels were 160 mg per deciliter (4.14 mmol per liter) or higher, high-density lipoprotein cholesterol levels were less than 40 mg per deciliter (1.03 mmol per liter), or triglyceride levels were 200 mg per deciliter (2.26 mmol per liter) or higher.

Participants were classified as having increased hypertension risk if they had a systolic blood pressure of 140 mmHg or higher or a diastolic blood pressure of 90 mmHg.

Participants were classified as having high-risk blood glucose levels if their blood hemoglobin A1C levels were 6% (42 mmol/mol) or higher.

Participants were classified as having heightened cardiometabolic risk if they exhibited high-risk lipid levels, high-risk blood glucose levels, or increased hypertension risk.

Latent BMI trajectories identification

Latent class mixture modelling (LCMM) was employed to investigate the longitudinal progression of BMI z-scores from birth to age 24 years, incorporating up to 14 BMI measurements per participant. We evaluated three polynomial functions for age (linear, quadratic, and cubic terms) when characterizing the longitudinal response of BMI z-score. Additionally, we incorporated sex as a covariate in the models. Each of these polynomial models, ranging from order 1 to 3, was respectively implemented within a one to seven-class solution. We initially assessed the predictive performance of different polynomial models by computing the mean absolute error loss of BMI z-scores using the 5-fold cross-validation technique. Subsequently, the optimal number of BMI z-score trajectories was determined based on a range of goodness-of-fit and discrimination indices, including the Bayesian Information Criteria (BIC), log-likelihood, and the values of mean posterior class membership probabilities. In addition to statistical parameters, clinical plausibility also played a crucial role in this decision-making process. The estimation of the models was done using the "lcmm" package in R (version 1.9.5).

Statistical analyses

For the liner regressions used to explore the associations of BMI trajectories with blood pressure, blood lipid, leukocytes, and HbA1c. The variables, which did not follow a normal distribution, will be transformed to normal distribution using the "bestNormalize" R package. We adjusted in model 1 for age and sex and in model 2 additionally adjusted for parental education, maternal smoking during pregnancy, maternal BMI at early

pregnancy, maternal hypertension, parity before the index person was born, cesarean section, and current smoking status. To investigate if current body fat composition contributed to the associations with cardiometabolic profiles, we performed a sensitivity analysis additionally adjusting for fat mass index in model 2.
